# Loss of electrical β-cell to δ-cell coupling underlies impaired hypoglycaemia-induced glucagon secretion in type-1 diabetes

**DOI:** 10.1038/s42255-024-01139-z

**Published:** 2024-09-23

**Authors:** Thomas G. Hill, Rui Gao, Anna Benrick, Lakshmi Kothegala, Nils Rorsman, Cristiano Santos, Samuel Acreman, Linford J. Briant, Haiqiang Dou, Nikhil R. Gandasi, Claudia Guida, Elizabeth Haythorne, Marsha Wallace, Jakob G. Knudsen, Caroline Miranda, Johan Tolö, Anne Clark, Lucy Davison, Joachim Størling, Andrei Tarasov, Frances M. Ashcroft, Patrik Rorsman, Quan Zhang

**Affiliations:** 1 Oxford Centre for Diabetes, Endocrinology and Metabolism, University of Oxford, Oxford, UK; 2 Metabolic Research Unit, Institute of Neuroscience and Physiology, University of Göteborg, Göteborg, Sweden; 3 Department of Developmental Biology and Genetics (DBG), Indian Institute of Science (IISc), Bengaluru, India; 4 Department of Physiology, Anatomy and Genetics, University of Oxford, Oxford, UK; 5 Nuffield Department of Clinical Medicine, University of Oxford, Roosevelt Drive, Oxford, UK; 6 The Royal Veterinary College, Hatfield, Hertfordshire, UK; 7 Section for Cell Biology and Physiology, Department of Biology, University of Copenhagen, Copenhagen, Denmark; 8 Steno Diabetes Center Copenhagen, Translational Type 1 Diabetes Research, Herlev, Denmark; 9 Biomedical Sciences Research Institute, School of Biomedical Sciences, Ulster University, Coleraine, Northern Ireland UK; 10 Oxford National Institute for Health Research, Biomedical Research Centre, Churchill Hospital, Oxford, UK; 11 Center for Neuroscience and Cell Biology (CNC), Centre for Innovative Biomedicine and Biotechnology (CIBB), University of Coimbra, Coimbra, Portugal

**Keywords:** Type 1 diabetes, Type 1 diabetes

## Abstract

Diabetes mellitus involves both insufficient insulin secretion and dysregulation of glucagon secretion^1^. In healthy people, a fall in plasma glucose stimulates glucagon release and thereby increases counter-regulatory hepatic glucose production. This response is absent in many patients with type-1 diabetes (T1D)^2^, which predisposes to severe hypoglycaemia that may be fatal and accounts for up to 10% of the mortality in patients with T1D^3^. In rats with chemically induced or autoimmune diabetes, counter-regulatory glucagon secretion can be restored by SSTR antagonists^4–7^ but both the underlying cellular mechanism and whether it can be extended to humans remain unestablished. Here, we show that glucagon secretion is not stimulated by low glucose in isolated human islets from donors with T1D, a defect recapitulated in non-obese diabetic mice with T1D. This occurs because of hypersecretion of somatostatin, leading to aberrant paracrine inhibition of glucagon secretion. Normally, K_ATP_ channel-dependent hyperpolarization of β-cells at low glucose extends into the δ-cells through gap junctions, culminating in suppression of action potential firing and inhibition of somatostatin secretion. This ‘electric brake’ is lost following autoimmune destruction of the β-cells, resulting in impaired counter-regulation. This scenario accounts for the clinical observation that residual β-cell function correlates with reduced hypoglycaemia risk^8^.

## Abstract (web summary)

Impaired somatostatin secretion owing to loss of β- to δ-cell electrical coupling underlies defective counter-regulatory glucagon secretion in type-1 diabetes.

## Main

**Extended Data Fig. 1:**
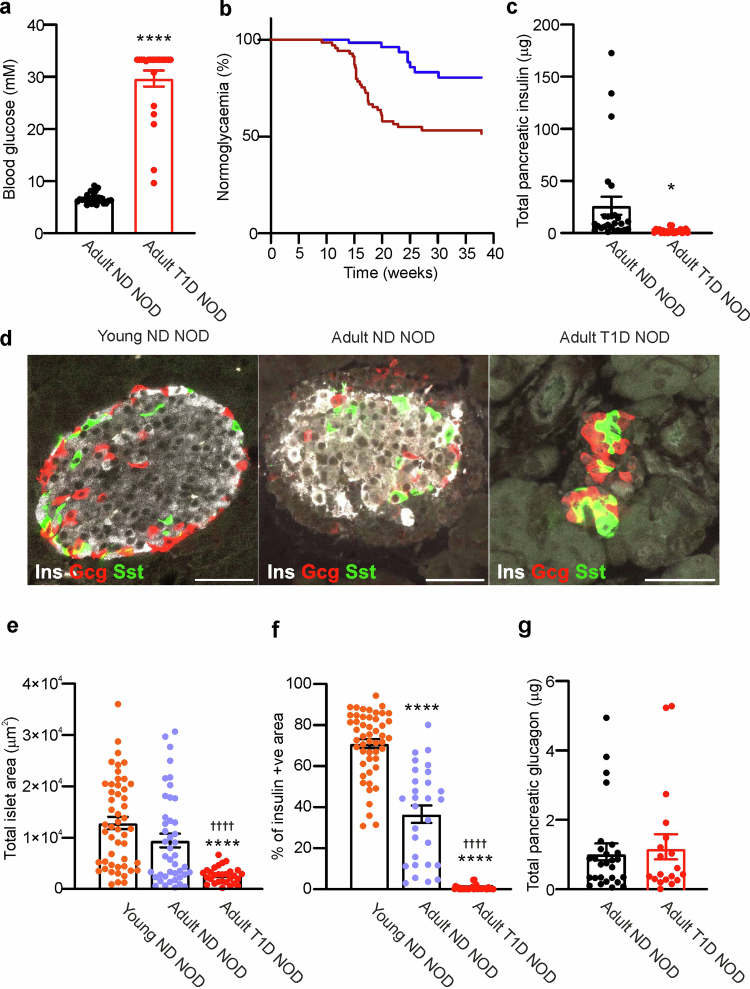
Impact of T1D on plasma glucose and pancreatic insulin and glucagon contents in NOD mice. **a**, Fed plasma glucose in adult NOD mice before (non-diabetic; ND, n = 27) and after onset of type-1 diabetes (T1D, n = 22). ****p = 3 × 10^−21^ vs ND. **b**, Kaplan Meier curves for the onset of T1D (loss of normoglycaemia) in NOD mice. Curves are based on 140 mice (70 males/70 females) of which 8 males (blue) and 31 females (red) developed T1D. Onset of diabetes displayed against age of mice (weeks). **c**, Whole-pancreas insulin content of adult ND (n = 22) and T1D NOD mice (n = 26). *p = 0.014 vs ND NOD mice. **d**, Representative immunohistochemistry of pancreatic islets from young ND NOD mice ( < 7 weeks old) and adult ( > 12 weeks old) ND and T1D NOD mice. Insulin (Ins) =white; glucagon (Gcg) =red; somatostatin (Sst) = green. Scale bar: 50 µm. Representative of 4 mice of each group. **e**, Islet area in young mice and adult mice with or without T1D. ****p < 0.0001 vs young ND NOD mice; ††††p < 0.0001 vs adult ND NOD mice. **f**, Relative β-cell area in young ND NOD mice and adult ND and T1D NOD mice. ****p < 0.0001 vs young ND NOD mice; ††††p < 0.0001 vs adult ND NOD mice. **g**, Whole-pancreas glucagon content of adult ND (n = 19) and T1D NOD mice (n = 27). In a, c and g, each dot corresponds to an individual animal. In (e-f), each dot represents an islet from 4 young ND NOD ( < 7 weeks old) or 4 adult ( > 12 weeks old) ND NOD mice and 4 adult T1D NOD mice. For each mouse, 3 representative sections were taken 100 µm apart. Rectangles and error bars behind data points represent mean values ± S.E.M. Two-sided Student’s t-test was used for (a and c) and one-way ANOVA with Tukey post hoc test was used for (e-f). Source data

We used non-obese diabetic (NOD) mice, a widely used polygenic mouse model of human T1D^9,10^, to explore why hypoglycaemia-induced glucagon secretion becomes impaired in diabetes. The onset of T1D correlates with an abrupt increase in random plasma glucose (Extended Data Fig. 1a). In a cohort of 140 mice, 31 out of 70 females and 8 out of 70 males developed T1D, with the onset in females preceding males by ~10 weeks (Extended Data Fig. 1b). T1D correlated with a 97% reduction in pancreatic insulin content, an ~80% decrease in islet area and nearly complete loss of insulin immunoreactivity and β-cell area whereas glucagon content remained unchanged (Extended Data Fig. 1c–g).

**Fig. 1:**
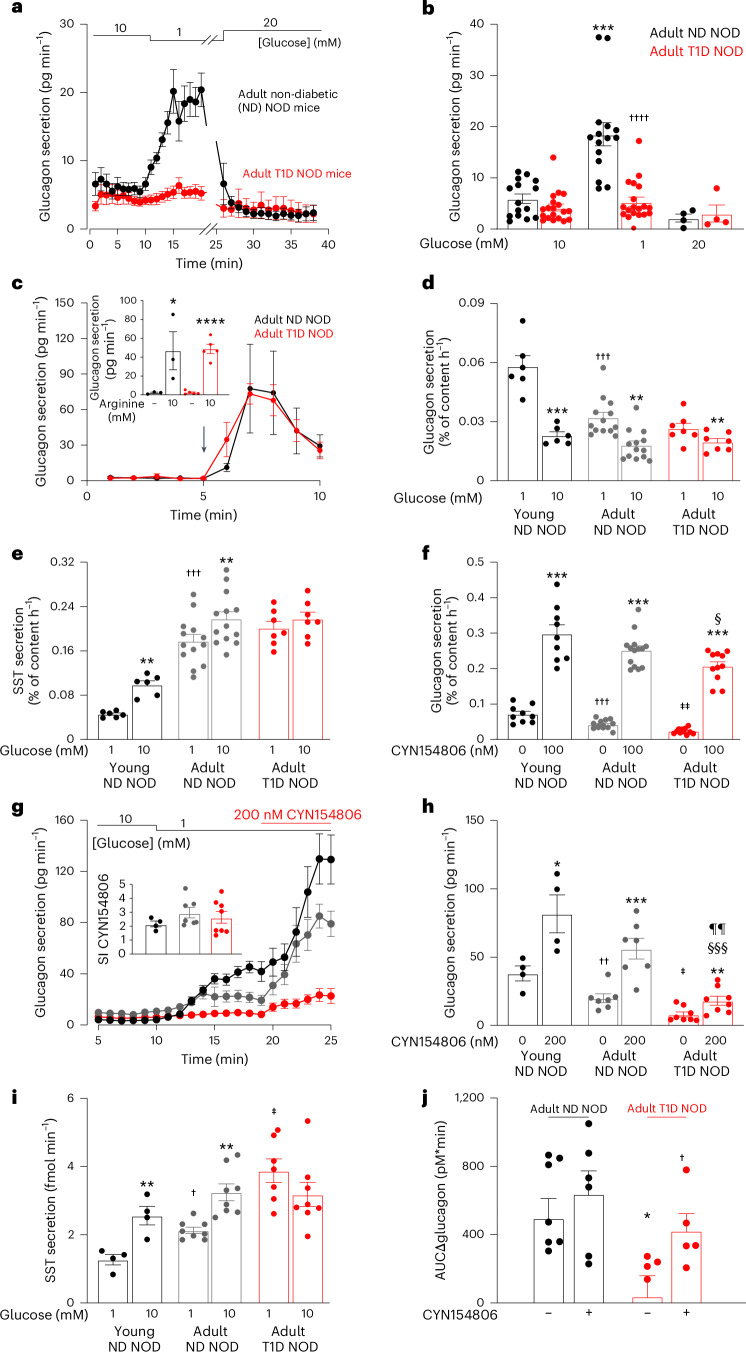
T1D abolishes hypoglycaemia-induced glucagon secretion owing to elevated somatostatin secretion. **a**, Glucagon secretion in perfused pancreases of adult ND or T1D NOD mice at indicated glucose concentrations (*n* = 15, 19 and 4 for 1, 10 and 20 mM glucose, respectively). **b**, Steady-state glucagon secretion in **a** under indicated conditions. ***P = 0.0003 vs 10 mM glucose; ††††*P* = 7.6 × 10^−5^ vs 1 mM glucose adult ND mice. **c**, As in **a** but the effect of arginine (arrow) in ND (*n* = 3) and T1D (*n* = 4 mice). Inset: mean glucagon secretion before and after addition of arginine in ND (black) and T1D (red) mice. *P = 0.046, ****P = 7.5 × 10^−6^ vs no arginine. **d**,**e**, Glucagon (**d**) and somatostatin (**e**) secretion at 1 mM and 10 mM glucose in young ND (*n* = 6 experiments with six mice; black), adult ND (*n* = 13 experiments with five mice; grey) and adult T1D islets (*n* = 7 experiments with five mice; red). **P < 0.01, ***P < 0.001 vs 1 mM glucose (same category); †††*P* < 0.001 vs 1 mM glucose young ND mice. **f**, As in **d** but testing CYN154806 (*n* = 9 experiments with six mice, *n* = 14 experiments with five mice and *n* = 5 experiments with five mice, respectively). ***P < 0.001 vs no CYN154806 (same category); †††*P* < 0.001 vs no CYN154806 young ND mice; ‡‡*P* < 0.01 vs no CYN154806 adult ND mice; ^§^*P* < 0.05 vs CYN154806 young mice. **g**–**i**, Glucagon secretion in pancreases of young ND (black, *n* = 4), adult ND (grey; *n* = 8) and T1D (red, *n* = 8) mice under indicated conditions (**g**), 5-min mean glucagon (**h**) or somatostatin secretion (**i**) under indicated conditions. *P < 0.05; **P < 0.01; ***P < 0.001 vs 1 mM glucose (same category); †*P* < 0.05; ††*P* < 0.01 vs 1 mM glucose young ND mice; ‡*P* < 0.05 vs 1 mM glucose adult ND mice; ^§§§^*P* < 0.001 vs CYN154806 young ND mice; ^¶¶^*P* = 0.003 vs CYN154806 adult ND mice. Inset in **g**: fold stimulation by CYN154806 (stimulation index, SI). **j**, Area under the curve (AUC) of plasma glucagon during insulin-induced hypoglycaemia in adult ND and T1D mice ±CYN154806. *P = 0.015 vs no CYN154806 adult ND NOD mice *n* = 6–8; †*P* = 0.038 vs T1D mice (*n* = 5–8). One-sided (in **c**) or two-sided unpaired *t*-test (in **b** and **j**); ANOVA with Dunnett’s post hoc (in **d**–**i**). Rectangles and error bars behind data points represent mean values ± s.e.m. SST, somatostatin. Source data

We compared low glucose-evoked glucagon secretion in NOD mice with and without T1D using the perfused mouse pancreas preparation (Fig. 1a,b). In normoglycaemic (non-diabetic; ND) NOD mice, lowering glucose from 10 mM to 1 mM robustly stimulated glucagon secretion by 348 ± 100% (*n* = 15). This stimulation was rapidly reversed when extracellular glucose was elevated to 20 mM. In age-matched hyperglycaemic (T1D) NOD mice, the stimulatory effect of low glucose on glucagon secretion was much weaker and limited to 34 ± 15% (*n* = 19; *P* < 0.01 vs ND). Notably, pancreases from T1D and ND NOD mice are equally responsive to 10 mM arginine (Fig. 1c). Therefore, the defective glucagon response to low glucose in T1D mice is not because they lack the capacity to produce or release the hormone. No differences were observed between male and female NOD mice with or without T1D.

**Extended Data Fig. 2:**
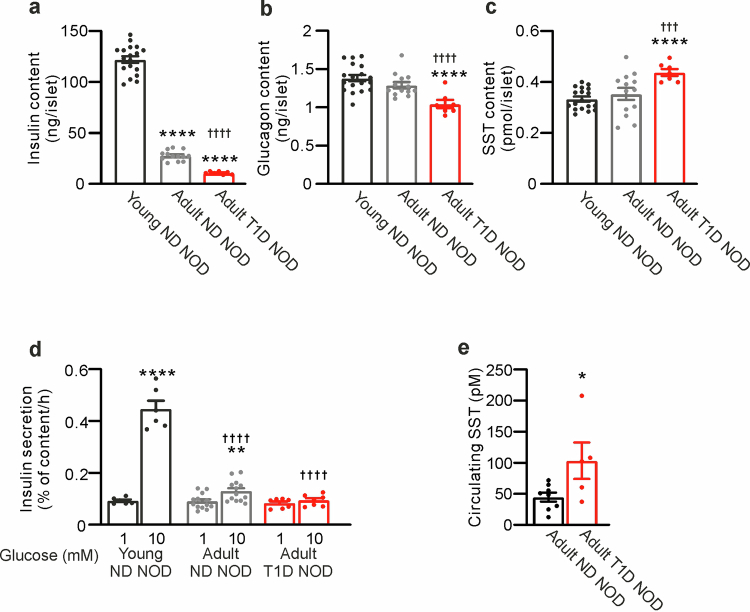
Effects of T1D on islet hormone contents and insulin secretion. **a-c**, Islet insulin (a), glucagon (b) and (c) somatostatin contents in young ND ( < 7 weeks old; n = 18 groups of 20 islets from 6 mice, black) and adult (n = 13 experiments with islets from 5 mice, >12 weeks old) ND (grey) and T1D NOD mice (n = 7 experiments with islets from 5 mice; red). ****p < 0.0001 vs young ND NOD islets; †††p = 0.0005, ††††p < 0.0001 vs adult ND NOD mice (one-way ANOVA with Tukey post hoc test). **d**, Insulin secretion at 1 and 10 mM glucose in young ND (n = 6 experiments with islets from 6 mice), adult ND (n = 13 experiments with islets from 5 mice) and adult T1D NOD mice (n = 7 experiments with islets from 5 mice). **p = 0.009 and ****p = 9.4 × 10^−7^ vs 1 mM glucose of the same category. ††††p = 7.6 × 10^−5^ (Adult ND) and ††††p = 2.6 × 10^−7^ (Adult T1D) vs 10 mM glucose in young NOD mice. Two-sided Student’s *t*-test. **e**, Circulating somatostatin in adult ND (n = 8) and T1D NOD mice (n = 5). *p = 0.0035 vs ND. Two-sided Student’s *t*-test. In dot plots, rectangles and error bars behind data points represent mean values ± S.E.M. Source data

We explored the underlying mechanisms in isolated islets from NOD mice. Data were obtained from young ND NOD mice (<7 weeks old), NOD mice with T1D (>12 weeks old) and their age-matched (>12 weeks old) ND NOD mice. T1D onset resulted in a dramatic decrease in islet insulin content but only marginally affected glucagon and somatostatin contents (Extended Data Fig. 2a–c), in agreement with human histological data^11^. Glucose-induced insulin secretion was reduced in age-matched ND and T1D adult mice compared to young ND mice (Extended Data Fig. 2d). There was a progressive decrease in glucagon secretion at 1 mM glucose in young ND, adult ND and T1D NOD mice that was paralleled by reduced glucagonostatic effect of 10 mM glucose (Fig. 1d). This correlated with a marked increase in somatostatin secretion at both 1 mM and 10 mM glucose (Fig. 1e). We reasoned that excessive somatostatin secretion (through paracrine inhibition) accounts for the failure of low glucose to stimulate glucagon secretion in T1D. We explored this hypothesis using the somatostatin receptor antagonist CYN154806. In isolated islets incubated at 1 mM glucose, CYN154806 produced a variable increase in glucagon secretion, ranging from ~300% in young ND NOD mice to >750% in adult T1D NOD mice (Fig. 1f). We confirmed these age-dependent changes in glucagon and somatostatin secretion and the effects of CYN154806 in NOD mice using the perfused pancreas preparation (Fig. 1g–i). Circulating somatostatin levels increased by ~100% after T1D onset in NOD mice (Extended Data Fig. 2e).

**Extended Data Fig. 3:**
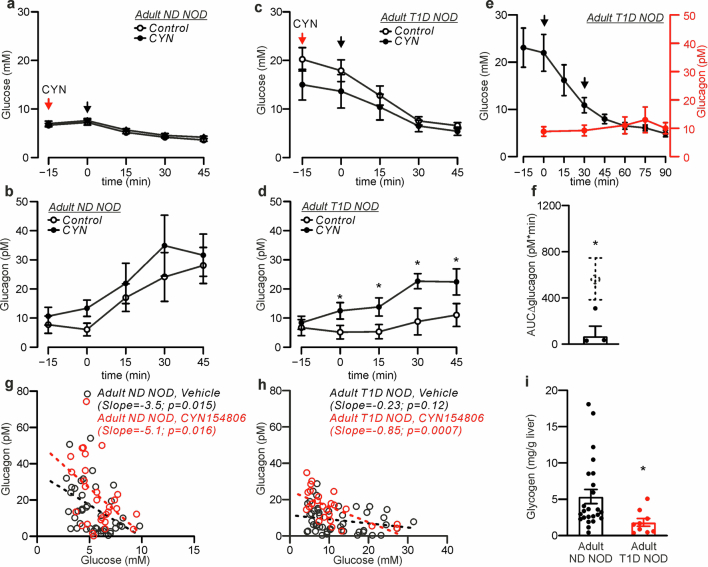
Impaired counter-regulation in NOD mice with T1D and correction by CYN154806. **a-b**, Changes in plasma glucose (a) and glucagon (b) in the absence (o; n = 8) and presence (•; n = 6) of CYN154806 in adult ND NOD mice. Insulin (0.75U/kg body weight) was injected at t = 0 (black arrow in a) and CYN154806 (0.5 mg/kg body weight) or vehicle was injected at t = −15 min (red arrow in a). **c-d**, As in (a-b) but using age-matched T1D NOD mice (n = 8 without and 5 with CYN154806). *p < 0.05 vs without CYN, two-sided Student’s *t*-test. **e**, Changes in plasma glucose (black) and glucagon (red) in T1D NOD mice (n = 3). Insulin (0.75U/kg body weight) was injected at t = 0 and 30 min (arrows). **f**, Plasma glucagon during hypoglycaemia induced by two consecutive insulin injections (0.75U/kg body weight) in T1D NOD mice (to lower plasma glucose to levels similar to those observed in ND mice). Data are expressed as the area under the curve (AUC) above basal (t = 30 min) during the 45 min following the second insulin injection (n = 3). For comparison, the responses in ND NOD mice are shown (mean value [dashed circle] ± S.E.M. [dashed lines], data taken from Fig. 1j); *p = 0.029 vs ND NOD mice, two-sided Student’s *t*-test. **g-h**, Relationship of plasma glucagon and glucose in ND (g) and T1D (h) NOD mice during insulin-induced hypoglycaemia in the absence (black) and presence (red) of CYN154806. Dashed lines mark the linear regression fittings of the data. Values of slopes (pM/mM) are given as well as the p values of the respective relationships obtained in the absence (vehicle) or presence of CYN154806. Statistical significance was evaluated using *t*-test. **i**, Glycogen content in livers from adult ND (n = 24) and T1D NOD (n = 9) mice. *p = 0.038 vs ND, two-sided Student’s *t*-test. In dot plots, rectangles and error bars behind data points represent mean values ± S.E.M. Source data

We compared plasma glucagon in ND and T1D NOD mice during insulin-induced hypoglycaemia in vivo in the absence and presence of CYN154806 (Fig. 1j and Extended Data Fig. 3a–f). In adult ND NOD mice, hypoglycaemia increased plasma glucagon by >300%, an effect almost abolished in age-matched T1D mice. CYN154806 did not affect hypoglycaemia-induced elevation of plasma glucagon in ND mice but had a marked effect in mice with T1D. Correlation analysis (Extended Data Fig. 3g–h) showed that glucagon secretion in T1D mice, unlike what was seen in ND NOD mice, was insensitive to changes in plasma glucose (*P* = 0.019 vs ND), a defect that was partially rectified by CYN154806 (*P* = 0.007 vs T1D without CYN154806). Hepatic glycogen content is much reduced in T1D mice (Extended Data Fig. 3i), which explains why the CYN154806-induced elevation of glucagon does not ameliorate hypoglycaemia in these mice (Extended Data Fig. 3c).

**Fig. 2:**
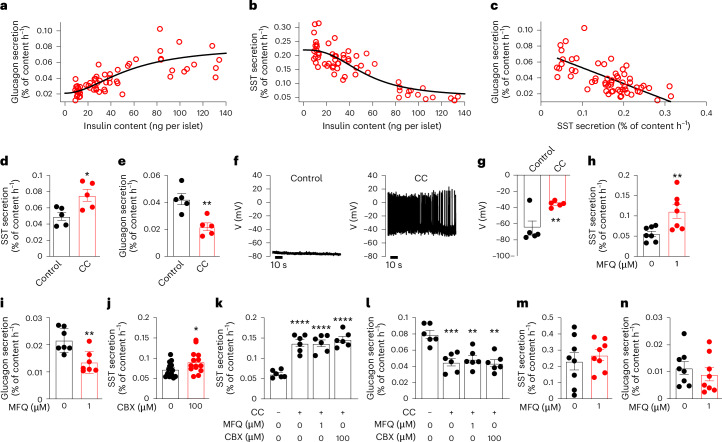
Loss of electrical coupling in T1D contributes to elevated intraislet somatostatin. **a**, Relationship between insulin content and glucagon secretion in isolated islets. The Hill equation (glucagon secretion = 1 ⁄ (1 + (EC_50_ / (insulin content)^*h*^) was fit to the data with an EC_50_ of 56 ± 9 nmol per islet and an *h* = 1.9 ± 0.5. **b**, As in **a**, but showing the relationship between insulin content and somatostatin secretion. EC_50_ = 53 ± 6 nmol per islet, *h* = −2.5 ± 0.5. **c**, Relationship between somatostatin secretion and glucagon secretion. Line: linear regression fitting of the data (*r* = 0.669; *P* < 0.0001). In **a**–**c**, data points (*n* = 60) represent groups of 20 islets (using 12 young ND, 10 adult ND and 10 T1D NOD mice). **d**,**e**, Somatostatin (**d**) and glucagon (**e**) secretion at 1 mM glucose in islets with or without 24 h CC pretreatment. *P = 0.02, **P = 0.004 vs 1 mM glucose alone (*n* = 5 using four mice). **f**, Membrane potential recordings from δ-cells in intact islets treated with saline (left) or CC (right). **g**, Most negative δ-cell (interspike) potential with or without CC pretreatment. **P = 0.0049 vs control (one-sided unpaired *t*-test). *n* = 5 cells in five islets from two mice. **h**,**i**, Somatostatin (**h**) and glucagon secretion (**i**) at 1 mM glucose with or without MFQ. **P = 0.0095 (in **h**); **P = 0.0038 (in **i**) vs 1 mM glucose alone (*n* = 7 using four mice). **j**, As in **h**, but testing the effect of CBX (100 µM). *P = 0.04 vs 1 mM glucose alone (*n* = 14 using four mice). **k**,**l**, Somatostatin (**k**) and glucagon secretion (**l**) at 1 mM glucose in islet pretreated or not with CC and in the presence of MFQ or CBX as indicated. **P < 0.01; ***P < 0.001; ****P < 0.0001 vs 1 mM glucose (*n* = 6 using four mice). **m**,**n**, Somatostatin (**m**) and glucagon (**n**) secretion at 1 mM glucose in islets from T1D NOD mice with or without MFQ as indicated (*n* = 5 using five T1D NOD mice; blood glucose >33 mM). Somatostatin secretion at 1 mM glucose alone was higher than in young ND NOD mice (*P* = 0.0115). Two-sided unpaired *t*-test was used in **d**, **e** and **h**–**n**. In dot plots, rectangles and error bars behind data points represent mean values ± s.e.m. CC, cytokine cocktail. Source data

We explored the relationship between insulin content (as a surrogate measure for β-cell mass) and somatostatin and glucagon secretion at 1 mM glucose in isolated NOD mouse islets at different stages of T1D progression (young ND, adult ND and adult T1D; see above). Glucagon secretion was reduced with decreasing islet insulin content, whereas somatostatin release increased (Fig. 2a,b). As a result, there was a negative correlation between somatostatin and glucagon secretion (Fig. 2c).

**Extended Data Fig. 4:**
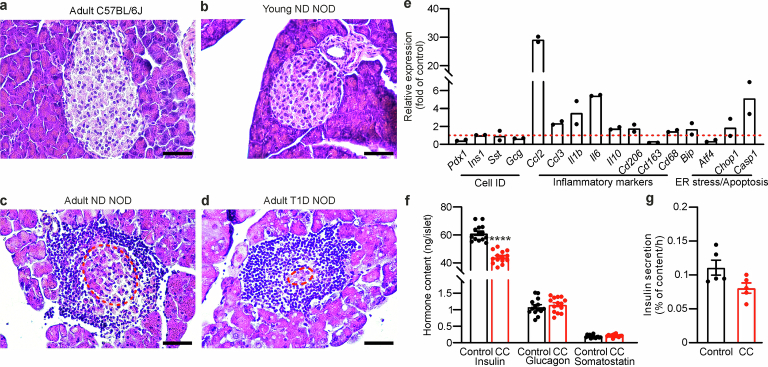
Immune cell infiltration in NOD islets and effects of cytokine treatment. **a-d**, Representative micrographs of mouse pancreases of wild type C57BL/6 J mice (16 weeks of age); young ND NOD mice ( < 7 weeks old; blood glucose: 5.6 mM), adult ND NOD mice ( > 12 weeks of age; blood glucose: 6.1 mM) and T1D NOD mice (blood glucose: 33 mM) as indicated. Scale bar: 50 μm. Representatives of 3 mice of each group. **e**, qPCR analyses of genes encoding islet cell-specific (*Ins1*, *Gcg*, *Sst*, *Pdx1*), inflammatory (*Ccl2-3*, *Il1b*, *Il6*, *Il10*, *Cd206*, *Cd163*, *Cd168*) and ER stress and apoptosis markers (*Bip*, *At4*, *Chop1*, *Casp1*) after treatment of mouse islets from C57BL/6 J mice for 24 h with a cytokine cocktail (CC). Expression has been normalised to control islets (no cytokines; indicated by dashed horizontal line) (n = 2 using islets from 6 mice). **f**, Islet insulin, glucagon and somatostatin contents measured with and without CC treatment as indicated. ****p = 1.9 × 10^−9^ vs control, two-sided Student’s *t*-test. **g**, Insulin secretion measured at 1 mM glucose in islets from C57BL/6 J mice with or without CC treatment as indicated (n = 5 experiments with islets from 4 mice). In dot plots, rectangles behind the data points represent the mean values ± S.E.M. Source data

Pancreases from NOD mice exhibit considerable immune cell infiltration of the islets even before detectable hyperglycaemia and destruction of the β-cells (Extended Data Fig. 4a–d). We emulated the humoral autoimmune attack on β-cells in T1D by treating isolated islets from healthy (C57BL/6J) mice in vitro for 24 h with a cytokine cocktail^12^. This treatment resulted in a dramatic upregulation of several inflammatory and apoptotic genes (Extended Data Fig. 4e) but islet hormone contents and gene expression were only marginally affected (if at all) (Extended Data Fig. 4e–g). However, somatostatin secretion was stimulated and glucagon secretion was inhibited (Fig. 2d,e), echoing the effects of T1D. Membrane potential recordings revealed that although δ-cells in control (vehicle-treated) islets exposed to 1 mM glucose invariably had a negative membrane potential and were electrically silent, all cells treated with the cytokine cocktail were depolarized and fired action potentials (Fig. 2f,g).

**Extended Data Fig. 5:**
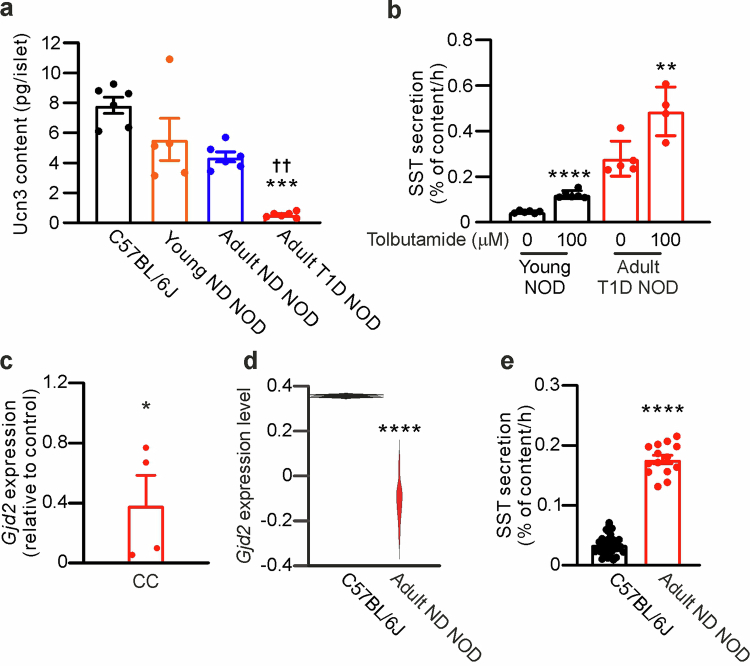
Impact of T1D on islet urocortin-3 contents and tolbutamide-induced somatostatin secretion. **a**, Urocortin-3 (ucn3) contents of islets from C57BL/6 J, young ND NOD, adult ND and adult T1D NOD mice. ***p = 0.004 vs young ND islets; ††p = 0.0036 vs adult ND islets (n = 5-6 experiments with islets from 4–6 mice). Urocortin-3 content in C57BL/6 J islets is also significantly higher than that in adult ND (p = 0.0093) and T1D NOD (p < 0.0001) mouse islets. Statistical significances were evaluated using one-way ANOVA with Tukey post hoc test. **b**, Effects of tolbutamide on somatostatin secretion in isolated islets from young ND and adult T1D mice. **p = 0.006 and ****p = 5.4 × 10^−7^ vs no tolbutamide in the respective group of islets (n = 4–6 experiments with islets from 4 mice for ND and T1D mice), one-sided Student’s *t*-test. **c**, Quantitative PCR of *Gjd2* expression in islets cultured with CC (n = 4 using islets from 6 mice). Data are normalised to *Gjd2* expression in islets incubated without CC. *p = 0.0489 vs control, two-sided Student’s *t*-test. **d**, Violin plot of average scaled expression of *Gjd2* from single-cell RNA sequencing data of δ-cells from wildtype (n = 298 cells; 2 samples; C57BL/6 J, black) and adult ND NOD (n = 1048 cells; 10 samples; red) mice. logFC = −0.16; ****p = 4.41 × 10^−6^, Wilcoxon Rank Sum test. **e**, Somatostatin secretion at 1 mM glucose in islets of wildtype (C57BL/6 J, black) and adult ND NOD (red) mice. ****p = 4.5 × 10^−25^ vs C57BL/6 J islets (n = 31 and 14 experiments using 4 mice for each group), two-sided Student’s *t*-test. In dot plots, rectangles and error bars behind data points represent mean values ± S.E.M. Source data

**Extended Data Fig. 6:**
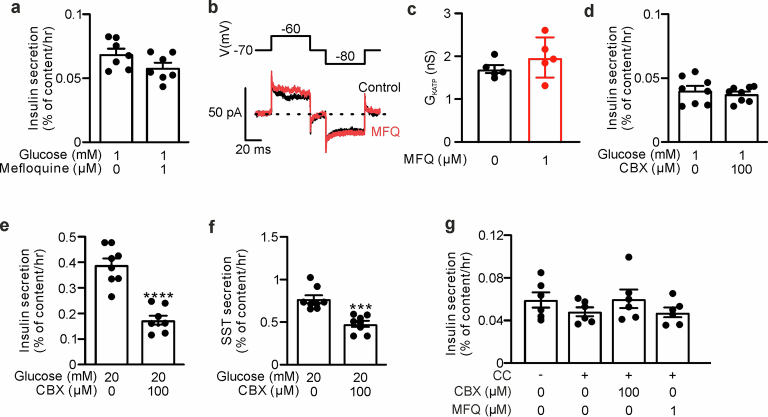
Effects of gap junction inhibitors on islet hormone release. **a**, Insulin secretion at 1 mM glucose in the absence and presence of the gap junction inhibitor mefloquine (1 μM) (n = 7 experiments with islets from 4 mice). **b-c**, K_ATP_ channel activity recorded in β-cells at 1 mM glucose in the absence (black) and presence (red) of 1 µM mefloquine (MFQ) as indicated (b). K_ATP_ channel activity is reported as conductance (G_KATP_), which was estimated from the current responses evoked by ±10 mV voltage pulses applied from a holding potential of −70 mV during perforated patch whole-cell measurements in β-cells in intact pancreatic islets (c) (n = 5 experiments). **d**, Insulin secretion from isolated islets from C57BL/6 J mice at 1 mM glucose in the absence or presence of the gap junction inhibitor carbenoxolone (CBX; 100 µM) as indicated. (n = 14 experiments with islets from 4 mice). **e-f**, Insulin (e) and somatostatin (f) secretion at 20 mM glucose in the absence and presence of CBX as indicated. ***p = 0.0002 and ****p = 8 × 10^−6^ vs no CBX (n = 8 from 4 mice), two-sided Student’s *t*-test. **g**, Insulin secretion at 1 mM glucose in islet pretreated or not with CC and in the presence of MFQ or CBX as indicated (n = 6 experiments from 4 mice). In dot plots, rectangles and error bars behind data points represent mean values ± S.E.M. Source data

**Extended Data Fig. 7:**
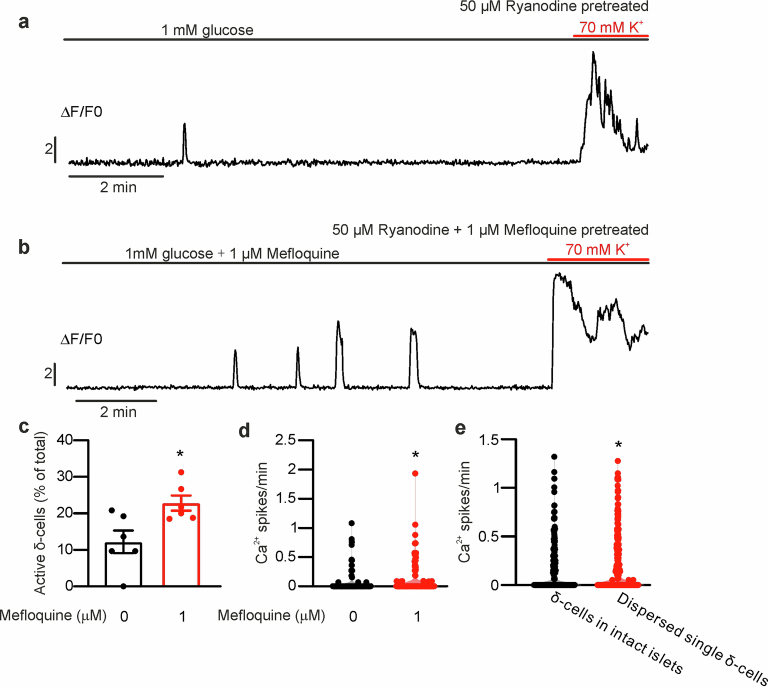
Electrical coupling and [Ca^2+^]_i_ in δ-cells. **a-b**, [Ca^2+^]_i_ oscillations recorded in single δ-cells in intact islets from Sst-GCaMP6f mice under control conditions (a) and after pretreatment (1 h) with 1 μM mefloquine (b). Experiments were concluded by increasing extracellular K^+^ to 70 mM to depolarise the δ-cells. Islets were pretreated with ryanodine (50 μM for 1 h) to minimise the contribution of intracellular Ca^2+^ release. (a, b) are each representative of 139–143 cells in 6 islets from 2 mice. **c**, Fraction of spontaneously active δ-cells within intact islets in the absence and presence of 1 µM mefloquine as indicated. *p = 0.019 vs no mefloquine (n = 6 islets obtained from 2 mice), two-sided Student’s *t*-test. **d**, Frequency of δ-cell [Ca^2+^]_i_ oscillations in the absence and presence of 1 μM mefloquine as indicated. *p = 0.042 (139 δ-cells in 6 islets from 2 mice without mefloquine and 143 δ-cells in 6 islets from 2 mice with mefloquine), two-sided Student’s *t*-test. **e**, Violin plots of δ-cell [Ca^2+^]_i_ oscillations in intact islets (240 δ-cells from 2 mice) and in dispersed islet cell preparations (380 δ-cells from 2 mice). *p = 0.038 vs intact islets, two-sided Student’s *t*-test. In dot plots, rectangles and error bars behind data points represent mean values ± S.E.M. Note stimulation of spontaneous [Ca^2+^]_i_ oscillations in the presence of the gap junction blocker mefloquine and that the depolarization-induced (high K^+^-induced) [Ca^2+^]_i_ increase was not affected by the inhibitor. Source data

We considered why somatostatin secretion is increased in T1D. Urocortin-3, co-released with insulin from β-cells, is a paracrine stimulator of somatostatin secretion^13^. In islets from NOD mice with T1D (that hypersecrete somatostatin), urocortin-3 content was reduced by >90% (Extended Data Fig. 5a). This finding, together with the observation that the ATP-regulated K^+^ (K_ATP_) channel blocker tolbutamide remained capable of stimulating somatostatin release in these urocortin-3-deficient islets (Extended Data Fig. 5b), indicates the existence of stimulatory mechanisms in addition to urocortin-3. Pancreatic β-cells and δ-cells are electrically coupled^14^. In agreement with a previous report^15^, cytokine cocktail pretreatment reduced the expression of *Gjd2* (Extended Data Fig. 5c), which encodes the gap junction protein connexin-36, expressed in both β-cells and δ-cells^15,16^. We also observed significant downregulation of *Gjd2* in δ-cells of pre-diabetic adult NOD mice (Extended Data Fig. 5d and M.W. and L.D., data in preparation) that correlated with increased somatostatin secretion (Extended Data Fig. 5e). We reasoned that membrane hyperpolarization of β-cells at low glucose, owing to the activation of K_ATP_ channels, normally spreads into the neighbouring δ-cells through the gap junctions. We tested the impact of β-cell to δ-cell electrical coupling on islet hormone release using the gap junction inhibitor mefloquine (MFQ)^17^. When applied at 1 mM glucose, MFQ (1 µM) stimulated somatostatin secretion and inhibited glucagon secretion (Fig. 2h,i) without affecting insulin secretion and β-cell K_ATP_ channel activity (Extended Data Fig. 6a–c), unlike what was reported for higher concentrations^18^. Likewise, the gap junction blocker carbenoxolone (CBX)^19^ also stimulated somatostatin secretion at 1 mM glucose (Fig. 2j), albeit less potently than MFQ (25% vs 99%), without affecting insulin secretion (Extended Data Fig. 6d). CBX has been reported to inhibit voltage-gated Ca^2+^ channels^20^. This effect may contribute to the 63% inhibition of glucose-induced insulin secretion that correlated with a 44% reduction of somatostatin secretion and explain the weaker stimulation by CBX at low glucose (Extended Data Fig. 6e,f). The weak effects of the gap junction blockers on insulin secretion at low glucose are consistent with previous reports in connexin-36-deficient (*Gjd2*^−/−^) mice^21–23^. Neither MFQ nor CBX exerted any additive effects on somatostatin and glucagon secretion in addition to those induced by cytokine cocktail (Fig. 2k,l) and did not affect insulin secretion (Extended Data Fig. 6g). MFQ also had no effects on somatostatin and glucagon secretion in islets from T1D NOD (Fig. 2m,n). Collectively, these findings suggest that the effects of both cytokine cocktail and T1D are mediated by inhibition of electrical coupling rather than unspecific effects (Extended Data Fig. 7). This conclusion does not exclude modulatory effects by factors released from the β-cells at high glucose and in healthy islets.

**Fig. 3:**
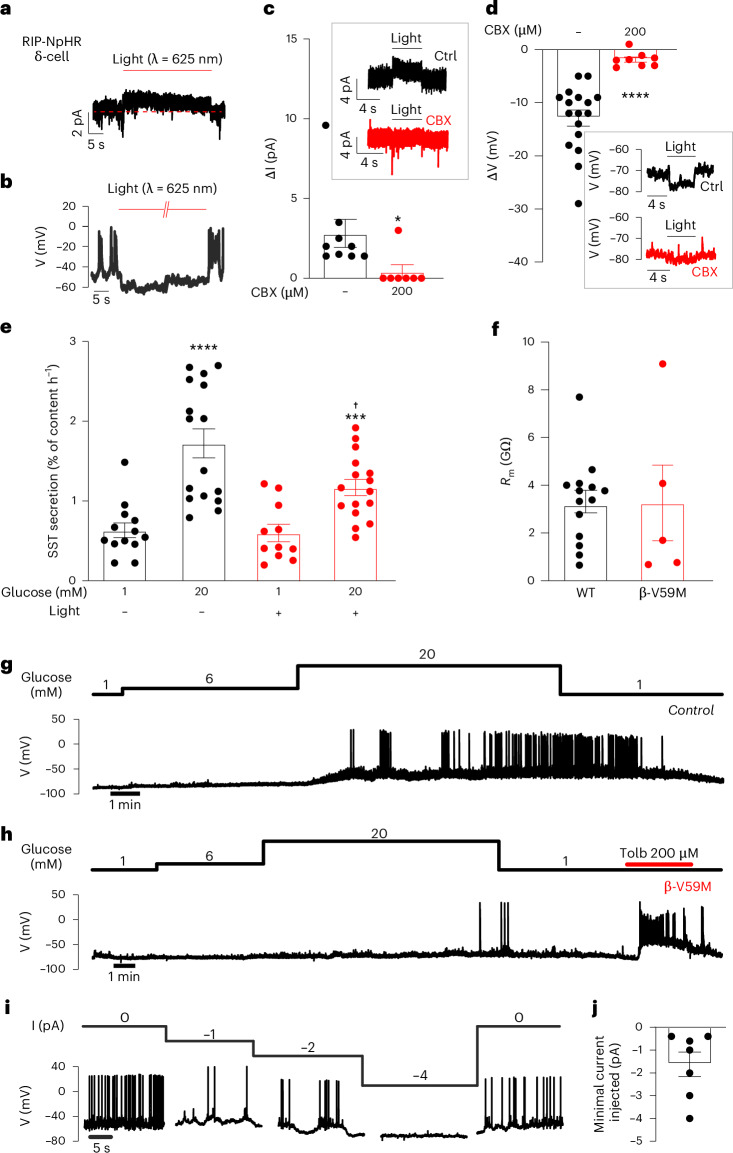
β-cell hyperpolarization via gap junctions prevents δ-cell electrical activity and somatostatin secretion. **a**,**b**, Examples of light-induced currents (**a**) and changes in membrane potential (**b**) in δ-cells of RIP-NpHR islets exposed to 10 mM glucose. Horizontal bars above the traces indicate onset of light activation (625 nm). In **a**, red dashed line indicates baseline. **c**, Amplitudes of light-induced currents (ΔI) in δ-cells ±200 μM CBX. Insets show δ-cell current excursions during optoactivation of NpHR in β-cells at 1 mM glucose in the absence (Ctrl) and presence of CBX. *P = 0.0328 vs no CBX (*n* = 7 cells without and *n* = 8 cells with CBX from seven mice). **d**, As in **c** but membrane potential changes (ΔV_m_) were measured. ****P = 1.4 × 10^−6^ (*n* = 17 cells without and *n* = 8 cells with CBX from seven mice). **e**, Somatostatin secretion in RIP-NpHR islets at 1 mM and 20 mM glucose ±NpHR activation in β-cells. ***P = 0.0009, ****P = 3.3 × 10^−5^ vs 1 mM glucose alone (±light activation); †*P* = 0.0126 vs 20 mM glucose without light activation (*n* = 11–16 experiments using islets from six mice). **f**, Membrane resistance in δ-cells at 1 mM glucose in islets from control and β-V59M mice. *P* = 0.96 vs control islets (*n* = 13 cells from eight control mice and *n* = 5 cells from four β-V59M mice). **g**, Electrical activity recorded from a δ-cell in a control islet at indicated glucose concentrations. Representative of five δ-cells from five mice. **h**, As in **g** but experiment performed in a δ-cell in an islet from hyperglycaemic β-V59M mouse 48 h after induction of the transgene expression (and diabetes) with tamoxifen. Representative of three δ-cells from three mice. **i**, Effects of injection of negative current (−1, −2 and −4 pA; top) on electrical activity (lower) in δ-cells at 10 mM glucose. **j**, Minimal current required to inhibit δ-cell electrical activity (*n* = 7 cells from five mice). Two-sided unpaired *t*-test was used in **c**–**f**. In dot plots, rectangles and error bars behind data points represent mean values ± s.e.m. WT, wild type. Source data

We explored the role of electrical coupling between β-cells and δ-cells further using an optogenetic mouse model expressing the light-gated chloride pump Np-halorhodopsin (NpHR)^24^ in β-cells (RIP-NpHR mice; Supplementary Fig. 1a,b). In β-cells in islets of RIP-NpHR mice exposed to 10 mM glucose, optoactivation of NpHR induced an outward current (112 ± 21 pA), hyperpolarized the cells by 78 ± 8 mV (*n* = 10 from three mice) and abolished action potential firing (Supplementary Fig. 1c,d). Optoactivation of NpHR in β-cells also promptly (time to peak: 24 ± 3 ms) induced an outward current of 3 ± 1 pA in neighbouring δ-cells, which was associated with 15 ± 1 mV (*n* = 11) hyperpolarization and suppression of δ-cell action potential firing (Fig. 3a,b). At 1 mM glucose (when β-cell secretion is suppressed), the repolarization of δ-cells was 10 ± 2 mV (*n* = 6; *P* = 0.12 vs 10 mM glucose), probably because of the reduced membrane resistance resulting from the activation of K_ATP_ channels at low glucose^25^. The persistence of the hyperpolarization at low glucose and the rapid onset are consistent with a gap-junction-mediated rather than paracrine mechanism. Indeed, the hyperpolarizing responses were strongly attenuated by CBX (Fig. 3c,d). We estimate (Equation 3.2. in ref. ^26^) the gap junctional conductance between β-cells and δ-cells to be 23 pS. This is only ~10% of that between pairs of β-cells^26–28^, which may reflect the lower expression of *Gjd2* in δ-cells^16^.

**Extended Data Fig. 8:**
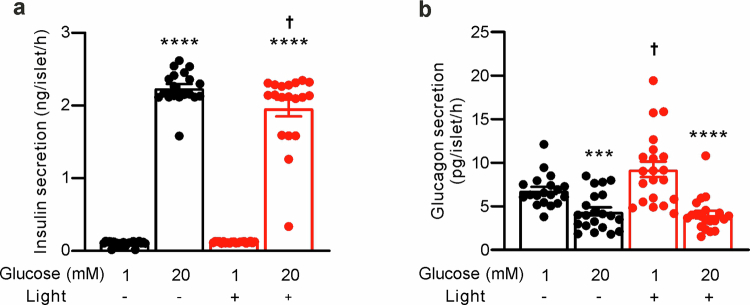
Effects of optoinhibition of β-cells on insulin and glucagon secretion. **a**, Insulin secretion at 1 and 20 mM glucose in islets from RIP-NpHR mice under control conditions (-) and during optoactivation (+) of NpHR in β-cells. ****p = 2 × 10^−34^ vs 1 mM glucose without NpHR optoactivation and ****p = 3.4 × 10^−19^ vs 1 mM glucose without NpHR optoactivation. †p = 0.0177 vs 20 mM glucose without NpHR activation (19–22 experiments with islets from 5 mice), two-sided Student’s *t*-test. **b**, As in (a) but glucagon secretion was measured. ***p = 0.0006 and ****p = 8.5 × 10^−6^ vs 1 mM glucose of respective group. †p = 0.0226 vs 1 mM glucose without NpHR optoactivation (19–21 experiments with islets from 5 mice), two-sided Student’s *t*-test. In dot plots, rectangles and error bars behind data points represent mean values ± S.E.M. Source data

Optoactivation of NpHR in β-cells inhibited glucose-induced insulin secretion but the effect was surprisingly weak (Extended Data Fig. 8a), possibly reflecting poor penetration of the light into deeper layers of the islet where most β-cells reside. Nevertheless, it strongly inhibited glucose-induced somatostatin secretion (Fig. 3e), confirming that a negative membrane potential in β-cell restricts somatostatin secretion. No detectable inhibitory effect was seen at 1 mM glucose, probably because δ-cell electrical activity and somatostatin secretion were already low. Nevertheless, glucagon secretion at 1 mM glucose was also stimulated during optoinhibition of β-cells (Extended Data Fig. 8b), an observation that may be related to the occurrence of spontaneous [Ca^2+^]_i_ oscillations at 1 mM glucose in a small subset of δ-cells^29^.

**Extended Data Fig. 9:**
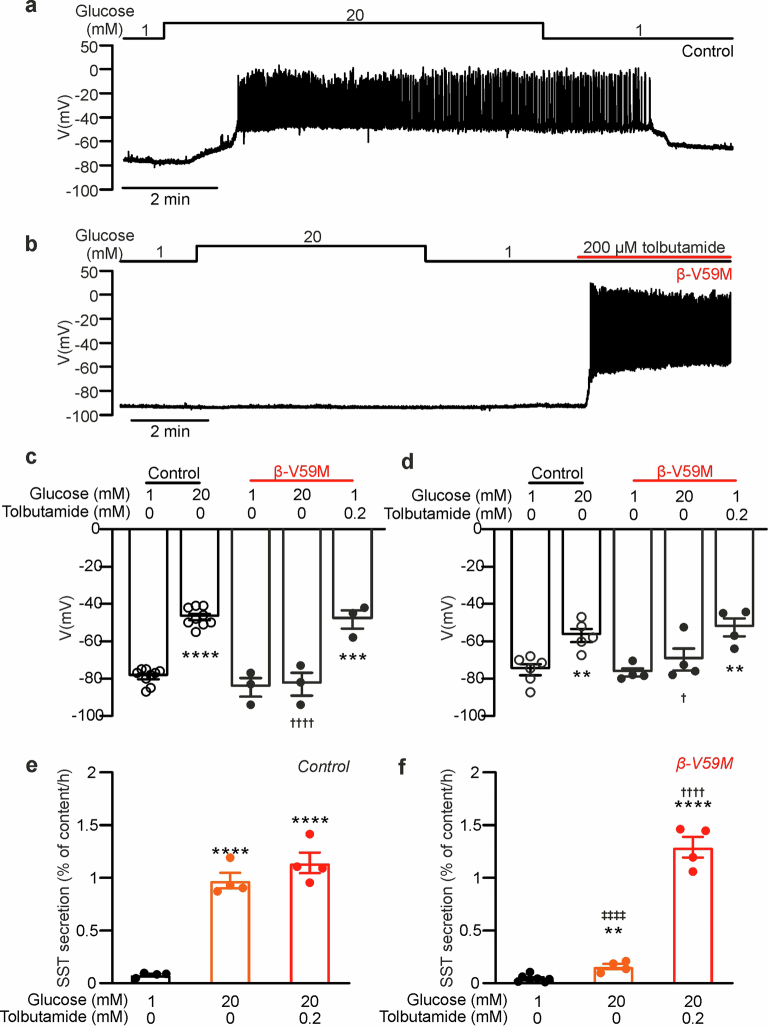
β-cell membrane potential controls somatostatin secretion. **a**, Electrical activity recorded from a β-cell in a control islet. Glucose was varied between 1 and 20 mM glucose as indicated. Representative of 9 δ-cells from 7 control mice. **b**, As in a but for a β-cell in an islet from hyperglycaemic β-V59M mouse 48 h after induction of the transgene expression with tamoxifen. Representative of 3 δ-cells from 3 mice. **c**, Membrane potential at 1 and 20 mM glucose in control (o) and induced β-cells in intact islets from β-V59M mice (•) (n = 9 β-cells from 7 control mice and 3 β-cells from 3 β-V59M mice).****p = 1.2 × 10^−7^ vs 1 mM glucose in control islets and ***p = 0.0003 vs 1 mM glucose in β-V59M islets (paired one-tailed Student’s t-test); ††††p = 4.2 × 10^−7^ vs 20 mM glucose in control β-cells (unpaired one-tailed *t*-test). **d**, As in (c) but data for δ-cells (n = 6 δ-cells from 6 control mice and 4 δ-cells from 4 β-V59M mice). **p = 0.009 vs 1 mM glucose in the respective mouse strain for 20 mM glucose and **p = 0.004 for the effect of tolbutamide on δ-cells in β-V59M islets (paired one-tailed *t*-test). †p = 0.04 vs 20 mM glucose in control δ-cells (unpaired one-tailed *t*-test). **e**, Somatostatin secretion in islets from control (non-induced) β-V59M mice under the conditions indicated. ****p = 1.9 × 10^−5^ vs 1 mM glucose for 20 mM glucose alone and ****p = 3.7 × 10^−5^ vs 1 mM glucose for 20 mM glucose with tolbutamide, two-sided Student’s *t*-test. Data from 4 mice. **f**, As in e but using islets from induced β-V59M mice (4 mice). **p = 0.0013, ****p = 3.6 × 10^−8^ vs 1 mM glucose; ††††p = 3 × 10^−5^ vs 20 mM glucose; ‡‡‡‡p = 4 × 10^−5^ vs 20 mM glucose in control islets, two-sided Student’s *t*-test. In dot plots, rectangles and error bars behind data points represent mean values ± S.E.M. Source data

If the membrane potential of β-cells influences δ-cells through gap junctions, then experimental paradigms that specifically prevent the glucose-induced depolarization in β-cells should interfere with electrical activity in neighbouring δ-cells. We tested this hypothesis using a mouse model that expresses the gain-of-function K_ATP_ channel V59M mutation in the β-cells^30^. We confirmed that β-cells in islets from β-V59M mice were refractory to high glucose but responsive to tolbutamide (Extended Data Fig. 9a–c). Somatostatin-secreting δ-cells are equipped with K_ATP_ channels that are identical to those in β-cells^31^. The δ-cell membrane resistance at 1 mM glucose was >3 GΩ in both diabetic β-V59M and control islets (Fig. 3f), confirming that the mutant channels are not expressed in δ-cells. In β-cells and α-cells, a membrane resistance this high is associated with action potential firing and stimulation of secretion^32,33^. The high input resistance combined with the fact that δ-cells are equipped with low-voltage activated Ca^2+^ channels^34^ should facilitate action potential firing. Yet δ-cells in intact islets are invariably electrically silent at 1 mM glucose (18 out of 18 cells). Control δ-cells consistently responded to 20 mM glucose with membrane depolarization and action potential firing (six out of six cells; Fig. 3g), whereas this was never the case in δ-cells from β-V59M mice (zero out of three cells; *P* = 0.0199 by Fisher’s exact test) but was observed following the addition of the K_ATP_ channel blocker tolbutamide (Fig. 3h and Extended Data Fig. 9d). Somatostatin secretion measurements in control and β-V59M islets corroborated the electrophysiology (Extended Data Fig. 9e–f). Given that δ-cells have very high input resistance at high glucose (16 ± 6 GΩ; *n* = 7), even minute currents entering through the gap junction will have dramatic effects on their membrane potential. We tested this process by injecting small hyperpolarizing currents into δ-cells (Fig. 3i). On average, an injection of −1.6 ± 0.5 pA was required to abolish glucose-induced action potential firing (Fig. 3j).

**Fig. 4:**
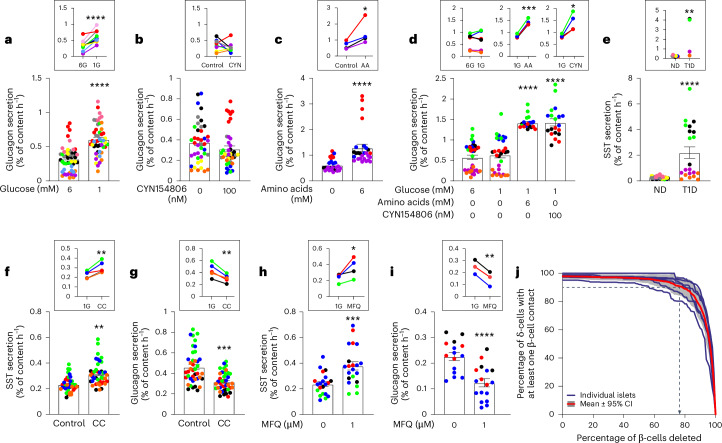
Effects of T1D on glucagon and somatostatin secretion in human islets. **a–c**, Glucagon secretion in human islets from ND donors at 6 mM and 1 mM glucose (6G and 1G) (*n* = 50, ten donors) (**a**), at 1 mM glucose ±100 nM CYN154806 (*n* = 41, eight donors) (**b**) or 6 mM amino acids (AA) (*n* = 27, four donors) (**c**). ****P = 6.676 × 10^−10^ (glucose) and ****P = 3.446*10^−5^ (AA). ****P = 7.7 × 10^−5^ (inset in **a**) and *P = 0.0362 (inset in **c**). **d**, Glucagon secretion in islets from donors with T1D at 6 mM and 1 mM glucose (*n* = 31–36 experiments, six donors) or ±CYN154806 or amino acids as indicated (*n* = 20 experiments, four donors). *P = 0.015, ***P = 0.00015, ****P < 0.0001. **e**, Somatostatin secretion at 1 mM glucose in islets from ND donors (*n* = 56, nine donors) and donors with T1D (n = 31, four donors). ****P = 1.8 × 10^−5^, **P = 0.004 (inset). **f**,**g**, Somatostatin (**f**) and glucagon (**g**) secretion at 1 mM glucose in islets from ND donors ±CC pretreatment for 24 h (*n* = 25, five donors). **P = 0.00105 (somatostatin), ***P = 0.00049 (glucagon) vs no CC. In insets, **P = 0.0024 (glucagon), **P = 0.0045 (somatostatin). **h**,**i**, Somatostatin (**h**) and glucagon (**i**) secretion at 1 mM glucose in islets from ND donors with or without MFQ (*n* = 21, four donors for somatostatin and *n* = 17, three donors for glucagon). ***P = 0.0005 (somatostatin), ****P = 9.39 × 10^−5^ (glucagon) vs control. In insets, *P = 0.035 (somatostatin) and **P = 0.002 (glucagon). **j**, Mathematical modelling of the fraction of δ-cells in contact with at least one β-cell expressed as a function of fraction β-cells deleted (%) in individual islets and mean of six islets. Shaded area, 95% confidence interval. Dotted lines, >90% of δ-cells are in contact with at least one β-cell until ~75% of β-cells have been removed. Rectangles and error bars behind data points represent mean values ± s.e.m. In **a**–**i**, different colours are used for each donor. Insets show mean data of the individual donors. Two-tailed unpaired *t*-test was used in **a**–**c** and **e**–**i**, one-way ANOVA with Tukey’s post hoc test was used in **d** and one-tailed paired *t*-test was used in the insets in **a**–**d** and **f**–**i**. One-tailed unpaired *t*-test was used in the inset of **e**. Source data

**Extended Data Fig. 10:**
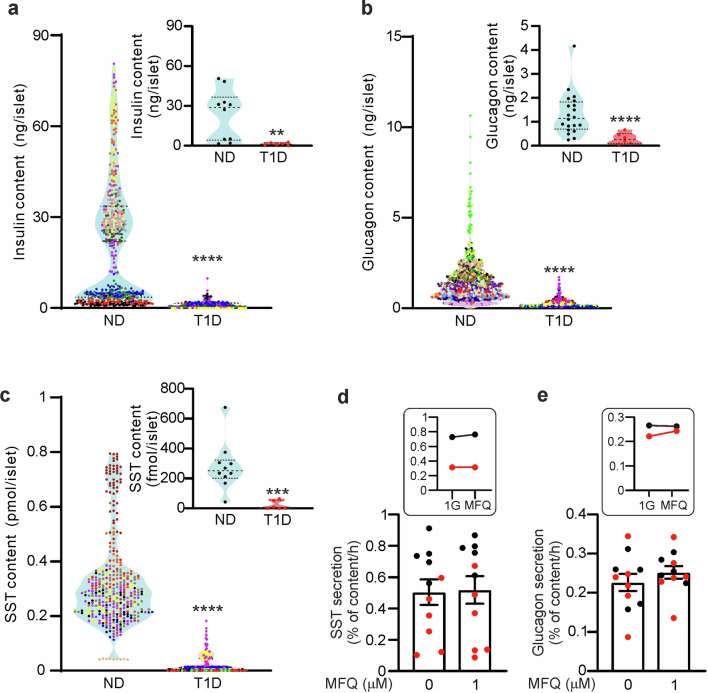
Islet hormone contents is reduced in T1D. **a-c**, Violin plot of islet insulin (a), glucagon (b) and somatostatin (c) content in islets from ND and T1D donors (10 ND and 6 T1D donors for insulin and somatostatin and 20 ND and 6 T1D donors for glucagon). Different colours used for data from each donor. Insets show mean values of individual donors. **p = 0.005, ***p = 0.0005 and ****p < 0.0001 vs ND, two-sided Student’s *t*-test. **d-e**, Somatostatin (d) and glucagon secretion (e) at 1 mM glucose in islets from T1D donors in the absence and presence of 1 µM MFQ as indicated. Insets show average data for the individual donors. In dot plots, rectangles and error bars behind data points represent mean values ± S.E.M. Source data

We extended our study to human pancreatic islets. In islets from organ donors without diabetes (that is, ND), lowering glucose from 6 mM to 1 mM consistently stimulated glucagon secretion by 147 ± 45% (*P* < 0.0001 by one-tailed *t*-test; Fig. 4a). In healthy islets exposed to 1 mM glucose, CYN154806 did not affect glucagon secretion, in agreement with previous work^35^, and the mean stimulatory effect was limited to 3 ± 24% (Fig. 4b). A cocktail of amino acids (6 mM total) increased glucagon secretion by 107 ± 23% (*P* < 0.01; Fig. 4c). In islets from donors with T1D, islet hormone contents were reduced (Extended Data Fig. 10a–c), glucagon secretion was not increased when glucose was lowered from 6 mM to 1 mM (7 ± 9%) but stimulated by amino acids (+68 ± 2%, *P* = 0.00015) or CYN154806 (+68 ± 15%, *P* = 0.015; Fig. 4d). Somatostatin secretion at 1 mM glucose dramatically elevated in T1D compared to ND islets (Fig. 4e). As in mouse islets, cytokine cocktail pretreatment stimulated somatostatin (34 ± 6%, *P* = 0.005) and inhibited glucagon secretion (−30 ± 2%, *P* = 0.002; Fig. 4f,g). MFQ also stimulated somatostatin (+52 ± 16, *P* = 0.035) and inhibited glucagon secretion (−41 ± 7, *P* = 0.0016; Fig. 4h,i). In two T1D preparations, MFQ had no effect on glucagon and somatostatin secretion at 1 mM glucose (Extended Data Fig. 10d,e), in agreement with the mouse data.

We finally consider the implications of these data for the management of patients with T1D. The failure of insulin-induced hypoglycaemia to increase plasma glucagon in T1D was first documented 50 years ago^2^. This defect, also seen in NOD mice^36^, was previously attributed to the loss of sympathetic innervation^36^; however, our finding that it persists in isolated mouse and human islets (where the nerves are severed) suggests that it also involves effects intrinsic to the islets and is mediated by somatostatin. The glucagonostatic action of somatostatin supersedes the stimulatory action of cAMP-increasing agents^37^ (like adrenaline^38^).

In healthy islets, electrical coupling allows β-cells to ‘share’ their K_ATP_ channels with δ-cells, as described previously for β-cell connections^39^. At low glucose, K_ATP_ channel activity is high in β-cells, resulting in membrane repolarization that spreads into δ-cells through gap junctions and leads to suppression of action potential firing and somatostatin release (see schematic in Supplementary Fig. 2a). At high glucose, β-cell depolarization extends into δ-cells and stimulates action potential firing and secretion (Supplementary Fig. 2b). Conversely, δ-cell electrical activity and somatostatin release at low glucose become stimulated in T1D following β-cell destruction and consequential removal of their hyperpolarizing influence. We modelled the relationship between β-cell number and β-cell to δ-cell connectivity in silico based on published architectures of human islets^40^. We tracked the fraction of δ-cells in direct contact with at least one β-cell as the β-cells were randomly removed. This remained >90% until >75% of the β-cells had been deleted (Fig. 4j).

Regulation of pancreatic islet hormone secretion by diffusible intercellular factors is well established^41^. The data we now present highlight an important, previously unrecognized, role of electrical signalling mediated by gap junctions between β-cells and δ-cells. They raise the interesting possibility that the protective effect of residual β-cell function is mediated by β-cell to δ-cell electrical coupling and β-cell hyperpolarization rather than secreted factors, leading to stimulation of somatostatin secretion and suppression of glucagon secretion. Notably, the risk of hypoglycaemia in T1D is reduced as long as circulating C-peptide levels (reflecting endogenous β-cell function) remain ≥15% of normal^8,42,43^, in good agreement with our in silico simulations. Our findings finally provide an experimental rationale for the use of agents that reduce somatostatin action or secretion as a means of restoring hypoglycaemia-induced glucagon secretion in T1D. Recent data suggest the clinical utility of such strategies^44^.

## Methods

### Ethics

All animal experiments were conducted in accordance with the UK Animals Scientific Procedures Act (1986) and University of Oxford and Gothenburg University ethical guidelines. The experiments were approved by the Oxford University Animal Welfare and Ethical Review Body and the Animal Welfare Body (Djurskyddsorganet) at the University of Gothenburg.

### Animals

NOD/ShiLtJ mice were purchased from Jackson Laboratories (Charles River, stock no. 001976). For brevity, we refer to hyperglycaemic NOD mice as T1D mice. Female NOD mice were used for most experiments because they develop T1D earlier than male mice^45^. Female NOD mice start developing T1D when they are >11 weeks old and plasma glucose abruptly increases from <10 mM to >30 mM, but up until 18 weeks of age, 30–50% of the mice remain normoglycaemic^45^ and are referred to as ‘non-diabetic’ (ND). Experiments were conducted in young (<7 weeks old) and adult (>12 weeks old) NOD mice with or without T1D. For studies in T1D NOD mice, age-matched and sex-matched ND NOD mice were used as controls. We acknowledge that some observations on the prevention of β-cell destruction in NOD mice have not been possible to translate to humans^46,47^, but here we focus on the impact of T1D on α-cell and δ-cell function, of which much less is known.

Sst-GCaMP6f mice (used in Extended Data Fig. 7) were generated by crossing floxed GCaMP6f mice (from JaxLab) and Sst-Cre mice. The Sst-Cre mice were generated as previously described^48^. Mice expressing the inducible Kir6.2-V59M transgene in insulin-secreting cells (used in Fig. 3 and Extended Data Fig. 9) were generated using a Cre-lox approach, as previously described^30^. Expression was induced in 12–14-week-old male and female mice with a single subcutaneous injection of 0.4 ml of 20 mg ml^−1^ tamoxifen in corn oil (Sigma-Aldrich). The successful induction of the transgene was confirmed by blood glucose measurements 48 h after injection of tamoxifen.

The RIP-NpHR mouse model (used in Fig. 3, Extended Data Fig. 8 and Supplementary Fig. 1) was generated by crossing RIP-Cre mice^49^ with Ai39 mice (Jax, no. 014539) that carry an improved halorhodopsin (HR) fused with a YFP, which are downstream of a *loxP*-flanked STOP cassette.

C57BL/6J mice were purchased from Envigo and were used in gene expression, hormone secretion, electrophysiology and histology studies when they were >15 weeks old.

For the NOD mice, blood glucose was monitored weekly. The mice were used within 48 h of developing hyperglycaemia. Blood glucose levels were measured from the tail vein using a Bayer Contour Next (Bayer) device.

Mice were housed in same-sex littermate groups of 2–8 animals, in a temperature-controlled and humidity-controlled room on a 12 h light–dark cycle (lights on at 07:00 h). Regular chow food (63% carbohydrate, 23% protein, 4% fat; Special Diet Services, RM3) was freely available. Water was available at all times.

### Human islets

Human pancreatic islets were isolated (with clinical consent) at the Nordic Islet Laboratory (Uppsala, Sweden), DRWF Human Islet Isolation Facility (Oxford, United Kingdom) and the Alberta Diabetes Institute IsletCore (Edmonton, Alberta, Canada). Human islet isolation was approved by the National Research Service, Oxford REC B (Oxford), Uppsala Regional Ethics Board (Uppsala) and Alberta Human Research Ethics Board (Pro00013094, Edmonton). This study was approved by the National Research Ethics Service, Oxford REC B (Ref: 09/H0/605/2).

Islets from the pancreases of 6 and 20 donors with and without T1D were used (Supplementary Table 1). Islets were isolated as previously described^35^. During the interval between islet isolation and the hormone secretion studies, islets were maintained in complete RPMI medium containing 5 mM glucose for up to 2 days before the experiments.

### Mouse islet isolation and tissue culture

Mice (7–20 weeks of age) of both sexes (only females for NOD mice) were killed by cervical dislocation. Liberase solution (Roche) was injected through the bile duct to inflate the pancreas. The pancreas was excised and digested in a water bath at 37 °C for 14 min. Islets were handpicked under a stereo microscope in Hanks’ balanced buffer. Islets from NOD mice were used after tissue culture in RPMI containing 10% FBS, 1% antibiotics and 10 mM glucose for up to 4 h. Islets from control mice (C57BL/6J) and β-V59M mice were used after overnight culture in RPMI supplemented with 5 mM glucose.

### Ca^2+^ imaging

For live-cell [Ca^2+^]_i_ imaging experiments, Sst-GCaMP6f islets were immobilized to a poly-l-lysine-coated coverslip fixed in a custom-built imaging chamber filled with Krebs–Ringer buffer (KRB) consisting of 140 mM NaCl, 3.6 mM KCl, 0.5 mM MgSO_4_, 2.6 mM CaCl_2_, 0.5 mM NaH_2_PO_4_, 2 mM NaHCO_3_, 5 mM HEPES and 6 mM glucose (pH 7.4 with NaOH). [Ca^2+^]_i_ imaging experiments were then performed using an inverted LSM 510 confocal microscope (Zeiss) controlled with ZEN Black (Zeiss), using a ×40/1.3 oil immersion objective. Time-lapse images were collected every 0.98 s with a frame size of 256 × 256 pixels, and the bath solution (KRB, glucose and pharmacological reagents with concentrations as indicated) was perfused at a rate of 0.4 ml min^−1^ at 37 °C. GCaMP6f was excited by an argon laser (488 nm) and emission was collected at 510 nm. [Ca^2+^]_i_ imaging videos were analysed using the Fiji imaging processing package (Version 1.54f, National Institutes of Health). The mean fluorescence (*F*) of each region of interest was normalized to the baseline signal (*F*_0_) and expressed as *F/F*_0_ before exporting into Clampfit (Version 9.2.0.11, Molecular Devices), where the baseline was corrected and [Ca^2+^]_i_ oscillation frequency was calculated.

### Antibody staining

Islets were fixed in 4% paraformaldehyde and then permeabilized with 0.3% Triton X-100 in phosphate-buffered saline (PBS) on ice for 30 min. Blocking of the tissue was done in PBS containing 5% goat serum for 30 min. Islets were then incubated with primary antibody for 2 h at room temperature or overnight at 4 °C, followed by incubation with secondary antibodies.

For fixed, paraffin-embedded tissue sections (5 µm), antigen retrieval (0.01 M sodium citrate, pH 6.0) was performed to unmask hidden antigenic sites. Non-specific blocking in swine serum (1:20 in PBS) was conducted, followed by immunofluorescent staining.

Antibodies used in this study included mouse anti-glucagon (clone name: K79bB10; AbCam, ab10988, 1:200; Sigma, G2654, 1:500), guinea pig anti-insulin (Europroxima, 2263B65-1, 1:200; ThermoFisher, PA1-26938, 1:500), goat anti-somatostatin (Santa Cruz, sc-7819, 1:100), rabbit anti-somatostatin (Dako, A0566, 1:200), rabbit anti-GFP (Abcam, ab6556, 1:2000), Alexa Fluor 594 donkey anti-mouse (Jackson Immune Laboratories, 715-587-003, 1:200), TRITC donkey anti-mouse (Thermo Fisher Scientific, A16071, 1:100), Alexa Fluor 594 goat anti-guinea pig (ThermoFisher, A-11076, 1:200), Alexa Fluor 546 donkey anti-goat (ThermoFisher, A11056, 1:100), Alexa Fluor 405 goat anti-mouse (ThermoFisher, A31553, 1:100), Alexa Fluor 488 goat anti-rabbit (ThermoFisher, A11008, 1:100) and Alexa Fluor 633 goat anti-guinea pig (ThermoFisher, A21105, 1:100).

The stained tissue was imaged in a confocal microscope (Bio-Rad Radiance) controlled by the LaserSharp2000 program (Bio-Rad). The islets were scanned in *Z*-stack imaging set to 1 µm per slice. Islet and hormone area was quantified using Fiji.

### Haematoxylin and eosin staining

Fixed, paraffin-embedded tissue sections were rehydrated in xylene followed by a descending alcohol series (100%, 90%, 75% and 0% (water)) for 3–5 min. Rehydrated sections were first incubated (5–8 min) in haematoxylin (H9627, Merck) to stain the nuclei, briefly rinsed in acid alcohol and then incubated (5 min) in eosin Y (E4009, Merck) to stain the cytoplasm and extracellular matrix. Stained sections were subsequently dehydrated in an ascending alcohol series followed by xylene (3–5 min) before coverslipping using DPX mounting medium (06522, Merck). Images were taken using a bright-field Nikon microscope and NIS-Elements software (Nikon).

### Plasma glucose and glucagon measurements

The mice were fasted for 2.5 h before the experiments. Tail vein blood glucose and glucagon levels were monitored using a glucometer (Contour Next, Bayer) before and during injection of insulin and other test substances as indicated. Hypoglycaemia was induced using a single intraperitoneal injection of insulin (0.75 U kg^−1^ in PBS, Actrapid, Novo Nordisk). The somatostatin antagonist CYN154806 (0.5 mg kg^−^^1^ body weight) or vehicle was injected 15 min before insulin. In a small group of T1D NOD mice, two consecutive insulin injections (0.75 U kg^−1^ for each injection; interval, 30 min) were used to produce stronger hypoglycaemia. Plasma glucagon was measured using ELISA (p/n 10-1281-01, Mercodia).

### Static hormone secretion

Islets (after 6–24 h in tissue culture) were pre-incubated in KRB containing 140 mM NaCl, 4.7 mM KCl, 2.5 mM MCaCl_2_, 1.1 mM KH_2_PO_4_, 1.2 mM MgSO_4_, 25 mM NaHCO_3_ and 10 mM HEPES (pH 7.4 with NaOH) supplemented with 3 mM glucose and 0.1% bovine serum albumin for 30 min at 37 °C. Sized-matched islets were dispensed in groups of 20 in test tubes with round bottoms containing 0.3 ml KRB supplemented with the indicated concentration of glucose for 1 h at 37 °C. Insulin and glucagon were measured by ELISA (Mercodia). Somatostatin was measured by radioimmunoassay (Diasource). Islet insulin, glucagon and somatostatin contents were measured after homogenization of the islets and sonication in acid ethanol. In some experiments, islets from C57BL/6J mice or human donors were treated with inflammatory cytokines for 24 h: 0.05 ng ml^−1^ IL-1β, 0.1 ng ml^−1^ TNF and 1 ng ml^−1^ IFNγ (PEPROTECH, 211-11B, 315-01 A and 315-05; BioLegend, 579402, 570102 and 570202). To ensure complete blockage of the gap junction connexin-36, MFQ (Merck Life Science, PHR1705) or CBX (Tocris, 3096) was applied in both pre-incubation and treatment buffers. MFQ was dissolved in dimethylsulfoxide and used at a concentration of 1 μM, while CBX was dissolved in water and used at a concentration of 100 μM. The cocktail of amino acids used consisted of 2 mM each of alanine, arginine and glutamine. The somatostatin receptor antagonist CYN154806 (Tocris, 1843) was dissolved in water and used at a concentration of 100–200 nM.

For studies on islets from RIP-NpHR mice, groups of 10 islets were transferred to 0.2 ml KRB in polystyrene tubes (55.484PS, Sarstedt) and placed in a 3D printed opaque box fitted with LEDs (LED591E, Thorlabs). The test tubes were illuminated from the bottom. Irradiance at the distance of the islets was measured using a power meter (PM100D/S120C, Thorlabs) to be 0.28 mW mm^−2^. Control experiments were performed identically except that no current was supplied to the LEDs. During the experiment, the boxes with the test tubes containing the islets were placed in a shaking incubator (ES-20, Biosan) pre-warmed to 37 °C. At the end of the 1 h incubation with (‘on’) or without (‘off’) illumination, the test tubes were placed on ice and samples removed for hormone measurements as described above.

### Whole-pancreas perfusion and pancreatic hormone extraction

Dynamic measurements of glucagon secretion were performed using in situ pancreas perfusion as previously described^50^. Throughout the experiment, the pancreas and the solutions infused were maintained at 37 °C. The infusion rate was adjusted for each animal according to body weight. The perfusate was collected from the portal vein every 1 min and stored on ice during the experiment and then frozen at −80 °C pending analysis. At the end of the experiments, the pancreases were resected and weighed. The tissues were then homogenized, sonicated in acid ethanol and stored at 4 °C for 1 week for total pancreas insulin and glucagon extraction and quantification.

### RNA extraction, cDNA synthesis and quantitative PCR

Total RNA from ~150 C57BL/6J mouse islets pretreated with or without a cytokine cocktail (IL-1β, INFγ and TNF) was extracted using Trizol (cat. no. T9424, Sigma-Aldrich) as previously described^51^. Potential contaminating DNA was removed by incubating the RNA for 20 min at 37 °C with DNase I (cat. no. 18068015, Invitrogen). The resulting RNA concentration and purity were quantified by measuring the absorbance at 260 nm (A260) and the A260/A280 ratio using a NanoDrop 1000 spectrophotometer (NanoDrop Products, Thermo Fisher), respectively. Approximately 200 ng of pure RNA was reverse transcribed into cDNA using the Applied Biosystem High-Capacity cDNA Reverse Transcription kit (cat. no. 4368814, Thermo Fisher), following the manufacturer’s instructions. The resulting cDNA samples were stored at −20 °C until used for quantitative PCR.

Quantitative PCR was conducted using a QuantStudio 7 Flex Real-Time PCR System (cat. no. 4486701, Applied Biosystems), whereby TaqMan Fast Universal Master Mix (cat. no. 4352042, Thermo Fisher) and designed TaqMan primer probes (1 µl) for the following genes of interest (Supplementary Table 2) were mixed with the cDNA (5 µl) of interest and RNase/DNase-free water (4 µl). Wells consisting of TaqMan primer and TaqMan Fast Universal Master Mix alone represented negative controls. The mRNA expression for the genes of interest was normalized to the mRNA expression of the housekeeping gene, *Actb*/*ACTB*. Relative quantification was assessed using the ΔΔCT method^52^. Agarose (1.5%) gel electrophoresis of the amplified PCR product was performed to validate the correct target amplicon size.

### Single-cell RNA sequencing

Single cells were processed on the 10× Genomics platform using kits pertaining to the V2 barcoding chemistry. Libraries were sequenced on Hiseq 4000 (Illumina) to an average of 96,000 reads per cell. Raw single-cell RNA sequencing reads were processed by Cell Ranger (version 7.1.0, 10X genomics) followed by quality control, cell clustering and transcriptomic analyses in R (version 4.0) using Seurat (v4).

### Glycogen measurements

Mouse livers were dissected and then frozen at −80 °C. Pieces of liver were cut, weighed, homogenized and resuspended in 200 μl water. The samples were then boiled for 10 min for enzyme inactivation and subsequently centrifuged at 18,000*g* at 4 °C. The supernatant was collected and the glycogen content was measured using a Glycogen Assay Kit (Abcam, ab65620). The optical density was read at 570 nm in a SpectraMax spectrophotometer.

### Urocortin-3 measurements

Total urocortin-3 content from 20 islets sonicated in 100 μl of acid ethanol was quantified by competitive ELISA (CED140Mu, Cloud-Clone Corporation) according to the manufacturer’s instructions.

### Electrophysiological recordings

Membrane potential recordings were measured in δ-cells within intact mouse islets as described previously^32^. Cell identity was established by electrophysiological fingerprinting^34^. All electrophysiological recordings were performed at ~34 °C. Membrane potential recordings were performed using an EPC-10 amplifier and Patch Master Software (HEKA Electronic) using the perforated patch whole-cell technique. Electrical access to the cell interior was obtained by amphotericin B added to the pipette-filling medium (final concentration, 150 μg ml^−1^). The extracellular solution contained 140 mM NaCl, 3.6 mM KCl, 1.3 mM CaCl_2_, 0.5 mM MgSO_4_, 5 mM NaHCO_3_, 0.5 mM NaH_2_PO_4_, 10 mM HEPES (pH 7.4 with NaOH) and glucose as indicated. The pipette solution was composed of 76 mM K_2_SO_4_, 10 mM KCl, 10 mM NaCl, 1 mM MgCl_2_ and 5 mM HEPES (pH 7.35 with KOH). Resting membrane resistance was estimated from the passive steady-state current responses (ΔI) by application of ±10 mV voltage pulses (ΔV) from a holding potential of −70 mV. The membrane resistance (*R*) was then calculated by Ohm’s law (*R* = ΔV/ΔI). In the current injection experiments, hyperpolarizing currents were injected during current-clamp measurements.

The optogenetics experiments were conducted in β-cells or δ-cells within intact RIP-NpHR islets using the perforated patch technique^32^. Optoactivation was effected by light (λ = 625 nm) applied using a computer-controlled LED light source (Mightex) through the objective (×60/1, Olympus) of the microscope. Irradiance measured at the tip of the objective using a power meter (PM100D/S120C, Thorlabs) was 23 mW mm^−2^. The LED was triggered from a controller with accompanying software (BLS-SA04-US, Mightex) and the light was continuous for the durations indicated. In the CBX experiments, islets were pre-incubated in 100 μM CBX for 1 h and the electrophysiological measurements were performed in the continued presence of CBX.

### Mathematical modelling

The determination of cellular contacts was conducted as previously described^14,40,53^. A δ-cell and a β-cell were considered as contacting if the location of their nuclei was less than a prespecified threshold derived from the length of projections observed in 3D electron microscopy images of human δ-cells (30 μm; ref. ^54^). β-cells were then randomly and systematically deleted from the architecture (mimicking the progression of T1D). On each deletion, the percentage of δ-cells with at least one β-cell contact was recalculated. This was repeated until all β-cells were removed from the islet architecture.

### Statistical analysis

In most instances, individual data points are shown. Data are reported as mean values ± s.e.m. for the indicated number of experiments using islets from multiple mice. For hormone release studies, isolated islets were pooled from 4–16 mice. These islets were then subdivided into groups of 20 size-matched islets. Each unique set of islets counted as an experiment to fully account for biological variability, with the experiments repeated on 2–4 days. This definition of an experiment is in accordance with published recommendations^55^ and 3R guidelines (https://eda.nc3rs.org.uk/experimental-design-unit). Data from electrophysiology, immunocytochemistry and Ca^2+^ imaging were treated analogously. Similarly, each unique group of human islets was treated as an experiment but mean data for each donor are also presented. The number of donors or mice used for each experimental series is indicated throughout.

Calcium imaging videos were analysed using Fiji and IgorPro (version 8, Wavemetrics). In brief, an in-house macro was used to auto-detect GCaMP6-expressing regions of interest representing individual δ-cells. The mean fluorescent intensities of these regions of interest were exported to IgorPro for individual wave plotting of each δ-cell. The mean fluorescent intensities were expressed as *F*/*F*_0_ and transformed using a Mexican hat filter and Fourier scaling for baseline correction. Area under the curve and spike frequency detection methods in IgorPro were then used to quantify these parameters for each δ-cell. The area under the curve and spike frequency data were compared back to the raw traces visualized in Fiji to confirm the accuracy and faithful representation of the raw data. Electrophysiology recordings were analysed using ClampFit (v.9.2.0.11, Molecular Devices), IgorPro (Wavemetrics) or Fitmaster (version 2x73.5, HEKA Electronic). For membrane potential recordings, data were exported from PulseFit (HEKA Electronic) as Igor Text files or ASCII files before being imported into IgorPro or Clampfit, respectively. In Clampfit, membrane potentials were manually measured and action potentials were detected using the ‘Template Search’ function under the ‘Event Detection’. The template of each recording was generated by averaging at least ten action potentials of the same cell. In IgorPro, action potential peak amplitude and interspike potentials were manually measured. Transmembrane currents recorded under voltage-clamp configuration were measured manually in Clampfit or Fitmaster.

Prism v.9.5 (GraphPad) and SPSS (IBM) software packages were used for the statistical analyses. Differences between two groups were assessed by one-tailed or two-tailed unpaired Student’s *t*-test (as indicated), and for differences between more groups, one-way ANOVA followed by a post hoc test was used. The significance of differences in the case of mixed within-samples between-samples design was tested using repeated-measures ANOVA with Dunnett’s post hoc.

### Reporting summary

Further information on research design is available in the Nature Portfolio Reporting Summary linked to this article.

## Supplementary information

Supplementary Information Supplementary Tables 1 and 2 and Supplementary Figs. 1 and 2.

Reporting Summary

## Source data

Source Data Fig. 1 Statistical source data.

Source Data Fig. 2 Statistical source data.

Source Data Fig. 3 Statistical source data.

Source Data Fig. 4 Statistical source data.

Source Data Extended Data Fig. 1 Statistical source data.

Source Data Extended Data Fig. 2 Statistical source data.

Source Data Extended Data Fig. 3 Statistical source data.

Source Data Extended Data Fig. 4 Statistical source data.

Source Data Extended Data Fig. 5 Statistical source data.

Source Data Extended Data Fig. 6 Statistical source data.

Source Data Extended Data Fig. 7 Statistical source data.

Source Data Extended Data Fig. 8 Statistical source data.

Source Data Extended Data Fig. 9 Statistical source data.

Source Data Extended Data Fig. 10 Statistical source data.

## Data Availability

All data generated and analysed during this study are included in this published article. The single-cell RNA sequencing data in Extended Data Fig. 5d was extracted from an unpublished dataset for one gene of interest. The dataset is not currently available for public access. Source data are provided with this paper.
