# Supplementary material for: Loss of electrical β-cell to δ-cell coupling underlies impaired hypoglycaemia-induced glucagon secretion in type-1 diabetes
Source: Nat Metab. 2024 Sep 23;6(11):2070–81. doi: 10.1038/s42255-024-01139-z (PMC11599053; doi:10.1038/s42255-024-01139-z)
Supplement: Supplementary file 1 — Supplementary Tables 1 and 2 and Supplementary Figs. 1 and 2. [file 42255_2024_1139_MOESM1_ESM.pdf]

nature metabolism

Supplementary information

https://doi.org/10.1038/s42255-024-01139-z

Loss of electrical β-cell to δ-cell coupling underlies impaired hypoglycaemia-induced glucagon secretion in type-1 diabetes

In the format provided by the authors and unedited

Supplementary Information

Supplementary Tables

Supplementary Table 1 │ Human islet donor characteristics.

**Islet preparation**	**1**	**2**	**3**	**4**	**5**	**6**	**7**
Donor age (years)	53	40	59	3	8	31	64
Donor sex (M/F)	M	F	F	M	F	M	M
Donor BMI (kg/m^2)	>25	>25	>25	<20	<20	>25	>25
Donor HbA_{1c}	6.0%	-	-	3.1%	4.8%	5.7%	6.7%
Islet isolation centre	Oxford	Oxford	Oxford	Alberta Isletcore, Canada	Alberta Isletcore, Canada	Alberta Isletcore, Canada	Alberta Isletcore, Canada
Donor history of T1D?	No	No	No	No	No	No	No
Donor cause of death	ICH	HBD	ICH	NDD	NDD		
Cold ischaemia time (h)	7	8	4	16.5	12.5	12	16
Estimated purity (%)	70	75	70-75	75	80	80	80
Total culture time (h)	NA	NA	NA	NA	NA	102	17

**Islet preparation**	**8**	**9**	**10**	**11**	**12**	**13**	**14**
Donor age (years)	65	32	75	64	55	42	50
Donor sex (M/F)	M	M	M	F	M	F	M
Donor BMI (kg/m^2)	>25	<25	<25	<25	>25	>25	>25
Donor HbA_{1c}	4.3%	4.5%	3.3%	4.5%	5.6%	-	5.7%
Islet isolation centre	Nordic Network for clinical islet transplan-tation	Alberta Isletcore, Canada	Nordic Network for clinical islet transplan-tation	Nordic Network for clinical islet transplan-tation	Nordic Network for clinical islet transplan-tation	Oxford	Nordic Network for clinical islet transplan-tation

Donor history of diabetes?	No	No	No	No	No	No	No
Donor cause of death	NA	NA	NA	NA	NA	NA	NA
Cold ischaemia time (h)	NA	NA	NA	20.39	NA	8	NA
Estimated purity (%)	13.35	NA	NA	NA	NA	70	NA
Total culture time (h)	NA	NA	NA	NA	NA	NA	NA

**Islet preparation**	**15**	**16**	**17**	**18**	**19**	**20**
Donor age (years)	52	55	62	28	65	27
Donor sex (M/F)	M	M	M	F	F	M
Donor BMI (kg/m^2)	>25	<25	<25	<25	>25	<25
Donor HbA_{1c}	3.7	3.6	5.4%	5.5%	5.7%	5.1%
Islet isolation centre	Nordic Network for clinical islet transplan-tation	Nordic Network for clinical islet transplan tation	Nordic Network for clinical islet transplan-tation	Alberta Isletcore, Canada	Alberta Isletcore, Canada	Alberta Isletcore, Canada
Donor history of diabetes?	No	No	No	No	No	No
Donor cause of death	NA	NA	NA	NA	NA	NA
Cold ischaemia time (h)	12	9	19.5	NA	NA	NA
Estimated purity (%)	NA	NA	NA	NA	NA	NA
Total culture time (h)	NA	NA	NA	NA	NA	NA

**Islet preparation**	**21**	**22**	**23**	**24**	**25**	**26**
Donor age (years)	70	23	38	44	57	42
Donor sex (M/F)	F	F	F	M	M	M
Donor BMI (kg/m^2)	<20	<25	>25	>25	>25	<25
Donor HbA_{1c}	5.5%	11.8%	10%	5.4%	8%	6.6%

Islet isolation centre	Nordic Network for clinical islet transplan-tation	Alberta Isletcore, Canada	Alberta Isletcore, Canada	Nordic Network for clinical islet transplan-tation	Alberta Isletcore, Canada	Nordic Network for clinical islet transplan-tation
Donor history of diabetes?	Yes	Yes	Yes	Yes	Yes	Yes
Donor cause of death	NA	NA	NA	NA	NA	NA
Cold ischaemia time (h)	6.06	NA	NA	NA	NA	10.02
Estimated purity (%)	NA	NA	10%	NA	NA	NA
Total culture time (h)	NA	NA	NA	NA	NA	NA

Supplementary Table 2 │ Primer probes used in qPCR.

Gene Symbol	Gene Name	Forward Primer (5' → 3')	Reverse Primer (5' → 3')
**Mouse**			
Pdx1	Pancreatic and duodenal homeobox-1	CGGACATCTCCCCATACG	AAAGGGAGCTGGACGCGG
Ins1	Insulin-1	GGCAGAGAGGAGGTACTTTGG	GGGTAGGAAGTGCACCAACA
Sst	Somatostatin	CCCAGACTCCGTCAGTTTCT	GGGCATCATTCTCTGTCTGG
Gcg	Glucagon	TCACAGGGCACATTCACCAG	CATCATGACGTTTGGCAATGTT
Cell-cell coupling/junction			
Gjd2	Gap junction protein delta-2	GGGGGAATGGACCATCTT	TCACCACCACAGTCAACAGG
Panx1	Pannexin-1	AGGCTGCCTTTGTGGATTC	TGGCAAACAGCAGTAGGATG
Panx2	Pannexin-2	CTGGTCACCCTGGTCTTCAC	ATCACGGGTGAAGTTGTGC
Inflammatory markers			
Ccl2	C-C motif chemokine ligand-2	GTTGGCTCAGCCAGATGCA	AGCCTACTCATTGGGATCATCTTG
Ccl3	C-C motif chemokine ligand-3	TGCCCTTGCTGTTCTTCTCT	GTGGAATCTTCCGGCTGTAG
Il1b	Interleukin-1 beta	GCAACTGTTCCTGAACTCAACT	ATCTTTTGGGGTCCGTCAACT
Il6	Interleukin-6	GCTACCAAACTGGATATAATCAGGA	CCAGGTAGCTATGGTACTCCAGAA
Il10	Interleukin-10	TCGGAAATGATCCAGTTTTAC	TCACTCTTCACCTGCTCCAC
Cd206	Mannose receptor C-type-1	GTTCACCTGGAGTGATGGTTCTC	AGGACATGCCAGGGTCACCTTT
Cd163	CD163 molecule	GGCTAGACGAAGTCATCTGCAC	CTTCGTTGGTCAGCCTCAGAGA
Cd68	Cluster of differentiation-68	GAAGCCGAATCAGCCTAGC	CAGCGTTACTATCCCGCTCT
Markers of ER stress			
Bip	Binding immunoglobulin protein	TTCAGCCAATTATCAGCAAACTCT	TTTTCTGATGTATCCTCTTCACCAGT
Atf4	Activating transcription factor-4	GGGTTCTGTCTTCCACTCCA	AGCAGCAGAGTCAGGCTTTC
Chop	C/EBP homologous protein	CCACCACACCTGAAAGCAGAA	AGGTGAAAGGCAGGGACTCA
Marker of cell death			
Casp1	Caspase-1	TCCGCGGTTGAATCCTTTTCAGA	ACCACAATTGCTGTGTGTGCGCA
Housekeeping gene			
Actb	B-actin	TGCTGTCCCTGTATGCCTCT	TTGATGTCACGCACGATTTC

Supplementary Figures

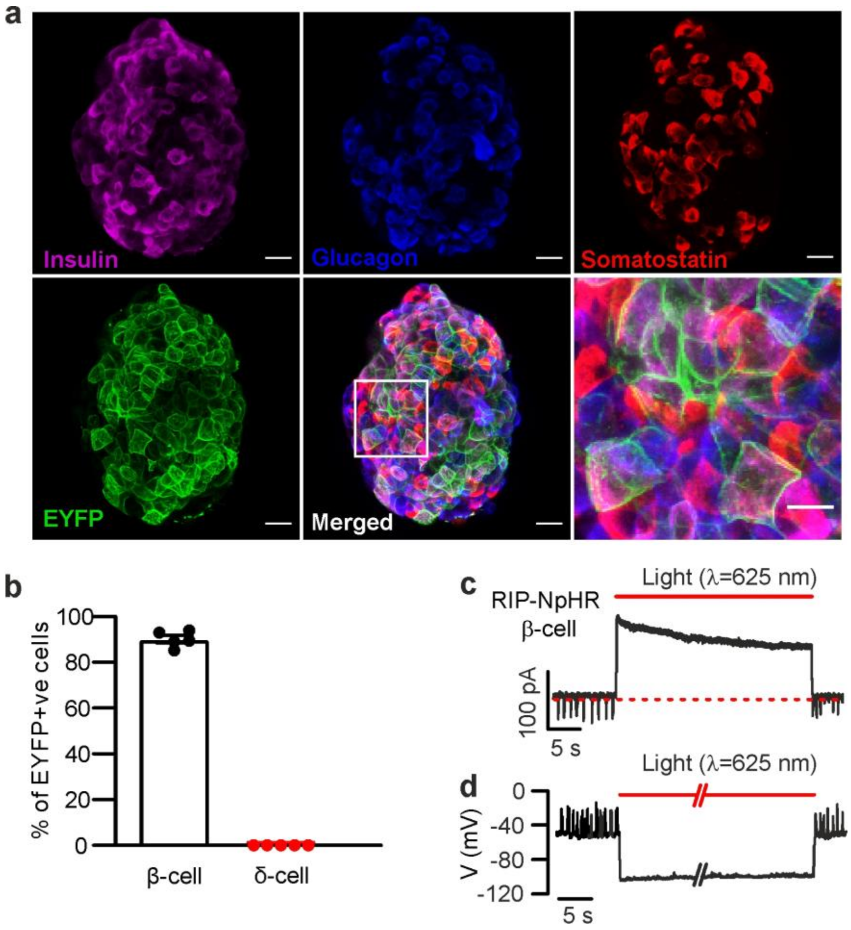

Supplementary Fig. 1 │ Generation of RIP-NpHR mice. a, Images of an NpHR islet stained for insulin (magenta), glucagon (blue), somatostatin (red) and EYFP (surrogate for NpHR, green). An area in the merged panel (white box) is displayed at greater magnification in the lower right panel. Scale bars=20 μm with the exception of the lower right panel (scale bar=10 μm). **b**, Percentage of EYFP+ cells in β- and δ-cells of NpHR islets (total of 497 insulin+ cells in 5 islets from 2 mice). **c-d**, Examples of light-induced currents (c) and changes in membrane potential (d) in β-cells of RIP-NpHR islets exposed to 10 mM glucose. Onset of light activation (625 nm) is indicated by horizontal bars above the traces. Red dashed line in (c) indicates the steady-state current level before light activation. Note that activation of NpHR repolarises the β-cell and abolishes action potential firing. In (c), the vertical downward deflections reflect action potential firing in neighbouring β-cells recorded via the gap junctions. Representative of 10 cells from 3 mice. In dot plots, rectangles and error bars behind data points represent mean values ± S.E.M.

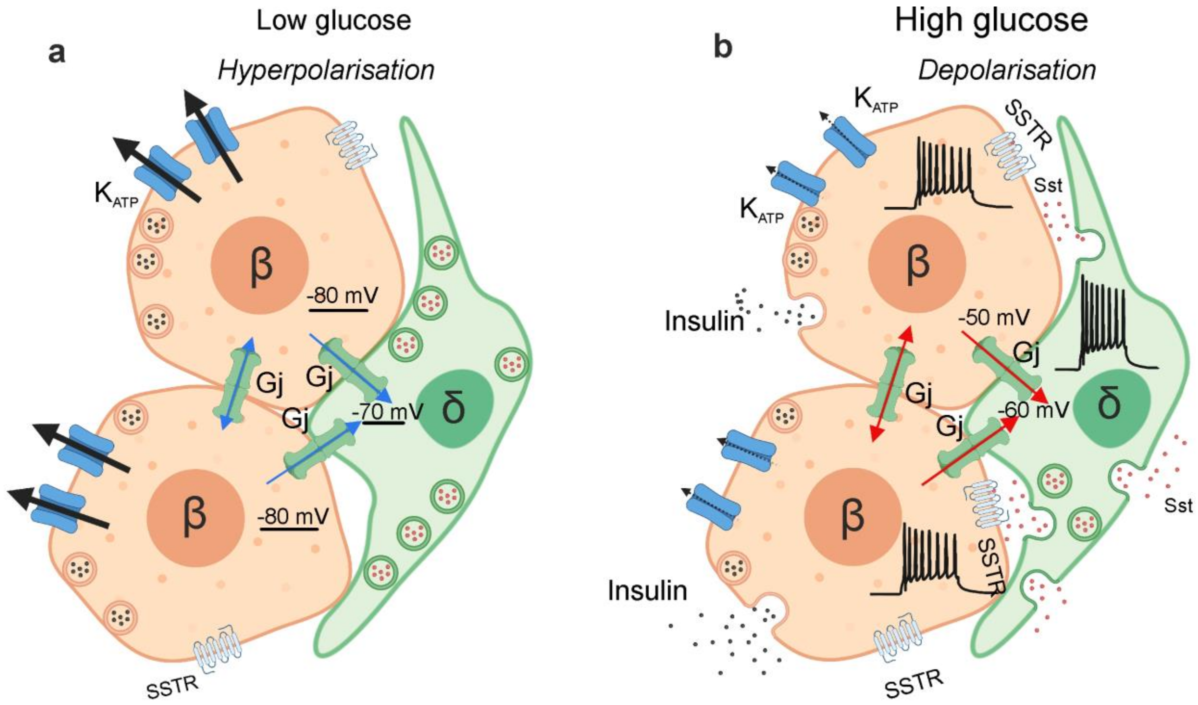

Supplementary Fig. 2 | Schematic of β-/δ-cell coupling in human islets. a-b, Schematic of regulation of δ-cell electrical activity via electrical coupling in healthy islets. At low glucose (a), K_{ATP} channel activity is high in the β-cells leading to a negative membrane potential (-80 mV) which is more negative than that of δ-cells (-70 mV). The voltage difference (ΔV) spreads via the gap junctions from the β- to δ-cells and injects a small hyperpolarizing current (blue) in the δ-cell, sufficient to suppress electrical activity. When glucose is increased (b), β-cell K_{ATP} channel activity is reduced, leading to membrane depolarization and action potential firing. This again spreads to the δ-cells but now gives rise to a depolarising current (red) and contributes to the stimulation of δ-cell electrical activity. The β-cells are also electrically coupled to each other, which serves to synchronize the electrical activity. Following the destruction of β-cells or loss of gap junction function, the δ-cells no longer experience the hyperpolarizing influence of β-cells at low glucose. The δ-cells therefore depolarise and start firing action potentials, accounting for the stimulation of somatostatin release. Created with BioRender.com.
