# Supplementary material for: Loss of electrical β-cell to δ-cell coupling underlies impaired hypoglycaemia-induced glucagon secretion in type-1 diabetes
Source: Nat Metab. 2024 Sep 23;6(11):2070–81. doi: 10.1038/s42255-024-01139-z (PMC11599053; doi:10.1038/s42255-024-01139-z)
Supplement: Supplementary file 2 — Reporting Summary [file 42255_2024_1139_MOESM2_ESM.pdf]

Corresponding author(s): Quan Zhang and Patrik Rorsman

Last updated by author(s): Aug 10, 2024

Reporting Summary

Nature Portfolio wishes to improve the reproducibility of the work that we publish. This form provides structure for consistency and transparency in reporting. For further information on Nature Portfolio policies, see our Editorial Policies and the Editorial Policy Checklist.

Statistics

For all statistical analyses, confirm that the following items are present in the figure legend, table legend, main text, or Methods section.

n/a	Confirmed	
☐	☒	The exact sample size (*n*) for each experimental group/condition, given as a discrete number and unit of measurement
☐	☒	A statement on whether measurements were taken from distinct samples or whether the same sample was measured repeatedly
☐	☒	The statistical test(s) used AND whether they are one- or two-sided<br>*Only common tests should be described solely by name; describe more complex techniques in the Methods section.*
☒	☐	A description of all covariates tested
☐	☒	A description of any assumptions or corrections, such as tests of normality and adjustment for multiple comparisons
☐	☒	A full description of the statistical parameters including central tendency (e.g. means) or other basic estimates (e.g. regression coefficient) AND variation (e.g. standard deviation) or associated estimates of uncertainty (e.g. confidence intervals)
☐	☒	For null hypothesis testing, the test statistic (e.g. *F*, *t*, *r*) with confidence intervals, effect sizes, degrees of freedom and *P* value noted<br>*Give P values as exact values whenever suitable.*
☒	☐	For Bayesian analysis, information on the choice of priors and Markov chain Monte Carlo settings
☒	☐	For hierarchical and complex designs, identification of the appropriate level for tests and full reporting of outcomes
☒	☐	Estimates of effect sizes (e.g. Cohen's *d*, Pearson's *r*), indicating how they were calculated

Our web collection on statistics for biologists contains articles on many of the points above.

Software and code

Policy information about availability of computer code

Data collection	Pulse (Version 8.80, HEKA Electronic), Patch Master (version 2x73.5, HEKA Electronic), ZEN Black (Version 6.0.0.303, Zeiss), µManager (Version 2.0.0, NIH), LaserSharp 2000 (Version 4.3, BioRad)
Data analysis	ClampFit (Version 9.2.0.11, Molecular Devices), Prism (Version 9.5.0, GraphPad), Fiji (ImageJ, Version 1.54f, NIH), PulseFit (Version 8.80, HEKA Electronic), Fit Master (version 2x73.5, HEKA Electronic), Cell Ranger (Version 7.1.0, 10X genomics), R Seurat (V4), MATLAB (Version R2023a, The MathWorks), IgorPro (version 8, Wavemetrics), SPSS (version 25, IBM).

For manuscripts utilizing custom algorithms or software that are central to the research but not yet described in published literature, software must be made available to editors and reviewers. We strongly encourage code deposition in a community repository (e.g. GitHub). See the Nature Portfolio guidelines for submitting code & software for further information.

Data

Policy information about availability of data

All manuscripts must include a data availability statement. This statement should provide the following information, where applicable:

- Accession codes, unique identifiers, or web links for publicly available datasets
- A description of any restrictions on data availability
- For clinical datasets or third party data, please ensure that the statement adheres to our policy

All data generated and analysed during this study are included in this published article. Source data are provided with this paper. The scRNAseq data in Extended Data Fig. 5d is extracted from an unpublished dataset for one gene of interest. The dataset is currently unavailable for public access.

Research involving human participants, their data, or biological material

Policy information about studies with human participants or human data. See also policy information about sex, gender (identity/presentation), and sexual orientation and race, ethnicity and racism.

Reporting on sex and gender	The study did not include human research participants. Human islets were obtained from anonymised and de-identified donors of both sexes (16 males and 10 females). All organ donors provided informed written consent for the use of human islets for research. Donor information is listed in Supplementary Table 1. No sex-based data analysis was performed due to the insufficient number of donors.
Reporting on race, ethnicity, or other socially relevant groupings	N/A
Population characteristics	Donor information is listed in Supplementary Table 1. Diabetes status was determined from patient records and HbA1c data.
Recruitment	No human research participants were recruited for this study.
Ethics oversight	Consent for organ donation for use in research was obtained from the donor prior to death or from relatives of the deceased donors conferred by the donor's physician and documented in their medical records. Human islet isolation was approved by the National Research Service, Oxford REC B (Oxford), Uppsala Regional Ethics Board (Nordic Network for Clinical Islet Transplantation) and Alberta Human Research Ethics Board (Pro00013094, ADI Isletcore, University of Alberta). This study was approved by the National Research Ethics Service, Oxford REC B (Ref: 09/H0/605/2).

Note that full information on the approval of the study protocol must also be provided in the manuscript.

Field-specific reporting

Please select the one below that is the best fit for your research. If you are not sure, read the appropriate sections before making your selection.

☒ Life sciences ☐ Behavioural & social sciences ☐ Ecological, evolutionary & environmental sciences

For a reference copy of the document with all sections, see nature.com/documents/nr-reporting-summary-flat.pdf

Life sciences study design

All studies must disclose on these points even when the disclosure is negative.

Sample size	Sample size for mice/mouse islets was determined with power calculation (G*Power3.1). Sample size was not determined for human islets, as it was however many samples were available.
Data exclusions	No data was excluded.
Replication	All experiments were successfully replicated in at least three independent biological individuals.
Randomization	For hormone secretion experiments using human islets, the same numbers of size-matched islets from the same donor were randomly allocated into different experimental groups. For hormone secretion experiments using mouse islets, the same numbers of size-matched islets from several mice were pooled before being randomly allocated into different experimental groups. For in vivo experiments, electrophysiology and Ca2+ imaging experiments, randomisation was not possible as mice or islets were treated differently during the experimental process.
Blinding	Blinding was not formally used, but assaying of the samples from the same type of experiments was processed together without knowledge of treatment groups (for example, ELISA measurements of hormones, all the samples with different treatments were read in the same plates using identical reagents and the same plate reader). It was impossible to blind when islets from diabetic animals or human donors were used due to their distinctive morphology. For human islets, the diabetic status was known upon arrival of the islets. Diabetic animals were identified (through blood tests) before experiments and therefore it was impossible to blind.

Reporting for specific materials, systems and methods

We require information from authors about some types of materials, experimental systems and methods used in many studies. Here, indicate whether each material, system or method listed is relevant to your study. If you are not sure if a list item applies to your research, read the appropriate section before selecting a response.

Materials & experimental systems

n/a	Involved in the study
☐	☒ Antibodies
☒	☐ Eukaryotic cell lines
☒	☐ Palaeontology and archaeology
☐	☒ Animals and other organisms
☒	☐ Clinical data
☒	☐ Dual use research of concern
☒	☐ Plants

Methods

n/a	Involved in the study
☒	☐ ChIP-seq
☒	☐ Flow cytometry
☒	☐ MRI-based neuroimaging

Antibodies

Antibodies used

Mouse monoclonal antibody to glucagon (Clone name: K79bB10; AbCam cat no. ab10988; 1:200 dilution, Sigma, G2654, 1:500 dilution), guinea-pig antibody to insulin (Europroxima, cat no. 2263B65-1, 1:200 dilution; ThermoFisher, cat no. PA1-26938, 1:500 dilution), goat antibody to somatostatin (Santa Cruz, cat no. sc-7819, 1:100 dilution), rabbit antibody to somatostatin (Dako, cat no. A0566, 1:200 dilution), rabbit antibody to GFP (Abcam, cat no. ab6556, 1:2000 dilution), Alexa Fluor 594 Donkey anti-mouse (Jackson immune laboratories, cat no. 715-587-003, 1:200 dilution), TRITC donkey anti-mouse (Thermo Fisher Scientific, cat no. A16071, 1:100 dilution), goat anti-guinea pig Alexa Fluor 594 (ThermoFisher, cat no. A-11076, 1:200 dilution), Alexa Fluor 546 donkey anti-goat (ThermoFisher, cat no. A11056, 1:100 dilution), Alexa Fluor 405 goat anti-mouse (ThermoFisher, cat no. A31553, 1:100 dilution,), Alexa Fluor 488 goat anti-rabbit (ThermoFisher, cat no. A11008, 1:100 dilution) and Alexa Fluor 633 goat anti-guinea pig (ThermoFisher, cat no. A21105, 1:100 dilution).

Validation

All primary antibodies are tested and characterized as specific in human tissues, by the manufacturers, and are widely cited.

1. Mouse monoclonal antibody to glucagon (Clone name: K79bB10; AbCam cat no. ab10988; 1:200 dilution, Sigma, G2654, 1:500 dilution).
https://scicrunch.org/ResourceWatch/Search?q=AB_297642
2. Guinea-pig antibody to insulin (Europroxima, cat no. 2263B65-1, 1:200 dilution; ThermoFisher, cat no. PA1-26938, 1:500 dilution).
https://www.antibodyregistry.org/AB_794668
3. Goat antibody to somatostatin (Santa Cruz, cat no. sc-7819, 1:100 dilution)
https://www.antibodyregistry.org/AB_2302603
4. Rabbit antibody to somatostatin (Dako, cat no. A0566, 1:200 dilution)
https://www.antibodyregistry.org/AB_2688022
5. Rabbit antibody to GFP (Abcam, cat no. ab6556, 1:2000 dilution)
https://www.antibodyregistry.org/AB_305564

Animals and other research organisms

Policy information about studies involving animals; ARRIVE guidelines recommended for reporting animal research, and Sex and Gender in Research

Laboratory animals

C57BL6J were purchased from Envigo.
Sst-GCaMP6f mice were generated by crossing floxed GCaMP6f mice (from JaxLab) and Sst-Cre mice.
The RIP-NpHR mouse model was generated by crossing RIP-Cre mice with Ai39 mice (Jax #014539) that carry an improved halorhodopsin (HR) fused with an YFP which are downstream of a loxP-flanked STOP cassette.
NOD/ShiLtj were purchased from Jackson Laboratories (via Charles River, stock no. 001976).
β-V59M mice Mice expressing the inducible Kir6.2-V59M transgene in insulin-secreting cells were generated using a Cre-lox approach.
For NOD/ShiLtj mice, young (<7wk old) and adult (>12 wk old) were used. For other strains, only adult animals (>12wk old) were used in the study.
Mice were housed in same-sex littermate groups of 2–8 animals, in a temperature- and humidity-controlled room on a 12-h light-dark cycle (lights on at 07:00). Regular chow food (63% carbohydrate, 23% protein, 4% fat; Special Diet Services, RM3) was freely available. Water was available at all times.

Wild animals

No wild animals were used in this study.

Reporting on sex

Female NOD mice were mainly used for experiments because they develop T1D earlier than male mice. Diabetes phenotype is identical in both male and female mice once they develop the disease.
Sex was otherwise not considered in the experimental design.

Field-collected samples	No field-collected samples were used in this study.
Ethics oversight	All animal experiments were conducted in accordance with the UK Animals Scientific Procedures Act (1986) and ethical guidelines of the universities of Oxford and Gothenburg and were approved by the Oxford University Animal Welfare and Ethical Review Body and the Animal Welfare Body (Djurskyddsorganet) at the University of Gothenburg.

Note that full information on the approval of the study protocol must also be provided in the manuscript.

Plants

Seed stocks	N/A
Novel plant genotypes	N/A
Authentication	N/A
